# Effects of exercise on cardiac structure and function in patients with type 2 diabetes: a narrative review of prospective imaging studies

**DOI:** 10.1007/s10554-025-03457-z

**Published:** 2025-07-19

**Authors:** Tin Gojevic, Felipe V. C. Machado, Youri Bekhuis, Guido Claessen, Veronique Cornelissen, Marieke De Craemer, Paul Dendale, Maarten Falter, Sara M. Ferreira, Lieven Herbots, Sarah Hoedemakers, Matthijs Michielsen, Mauricio Milani, Jan Verwerft, Dominique Hansen

**Affiliations:** 1 Rehabilitation Research Institute (REVAL), Hasselt University, Hasselt, Belgium; 2 Faculty of Rehabilitation Sciences, Hasselt University, Hasselt, Belgium; 3 Faculty of Movement and Rehabilitation Sciences, Department of Rehabilitation Sciences, KU Leuven, Leuven, Belgium; 4 Faculty of Medicine and Health Sciences, Department of Rehabilitation Sciences, Ghent University, Ghent, Belgium; 5 Department of Cardiology and Jessa & Science, Jessa Hospital, Hasselt, Belgium; 6 Department of Cardiology, University Hospital of Leuven, Leuven, Belgium; 7 Department of Cardiovascular Diseases, UZ Leuven, Leuven, Belgium; 8 Faculty of Medicine and Life Sciences/LCRC, UHasselt, Diepenbeek, Belgium; 9 Graduate Programme in Health Sciences and Technologies, University of Brasilia (UnB), Brasilia, DF Brazil; 10 Faculty of Rehabilitation Sciences, Hasselt University, Wetenschapspark 7, Hasselt, Diepenbeek, 3590 Belgium

**Keywords:** Exercise, Training, Type 2 diabetes, Heart failure, Cardiac dysfunction, Diabetic cardiomyopathy

## Abstract

Around 30% of patients with type 2 diabetes(T2D) develop heart failure and this leads to poor prognosis. Treatment with exercise intervention can improve left cardiac function at rest in T2D, but its effects on subclinical HF markers, maximal cardiac function during exercise, and right cardiac function are unknown. This review aimed to synthesize the effects of exercise on cardiac structure and function in patients with T2D from prospective cardiac imaging trials. The secondary aim was to address the methodological shortcomings of previous studies and map future avenues. Our synthesis showed that exercise interventions can improve left ventricular structure and systolic and diastolic function at rest and during exercise, and right ventricular systolic function at rest in patients with T2D. Study shortcomings were inadequate reporting of randomization, concealment, between-group point estimates, intervention adherence, attendance and adverse events. Future studies should investigate sex- and phenotype-specific effects of exercise on the left and especially right heart during rest and peak exercise, determine the optimal exercise intensity, duration and volume for inducing cardiac changes, and determine if cardiac changes translate to a long-term prognosis.

**Supplementary Information:**

The online version contains supplementary material available at 10.1007/s10554-025-03457-z.

## Introduction

In the early 1970 s, postmortem examinations of patients with type 2 diabetes (T2D) for the first time revealed the development of heart failure (HF) without accompanying hypertension, myocardial ischemia, or congenital or valvular heart disease [1]. This signaled a causal relationship between T2D and HF, giving rise to the new condition called diabetic cardiomyopathy [1]. Diabetic cardiomyopathy is characterized by negative structural, functional and metabolic cardiac remodelling that increases the risk of HF [2, 3]. About 30% of patients with T2D have HF [4–6], and 28% of patients with T2D receive HF diagnosis in the first five years of follow-up [7]. The coexistence of T2D and HF exacerbates the risk of adverse events and death [7–9], emphasizing preventive needs.

Exercise training is a widely-recognized intervention for improving glycemic profile, cardiovascular risk factors and cardiorespiratory fitness in patients with T2D [10–12]. Cardiac imaging studies showed that exercise intervention, next to pharmacological agents [13, 14], weight loss procedures [15], and dietary interventions [16], protects the heart of a patient with T2D [17, 18]. A meta-analysis of the effects of exercise on the heart in patients with type 2 diabetes showed that exercise intervention improves imaging markers of subclinical HF by increasing early diastolic tissue velocity and global longitudinal strain [18]. However, this meta-analysis did not include data on left atrial function, and right atrial and ventricular function, which are also negatively affected by T2D [19]. Additionally, the meta-analysis did not include cardiac imaging markers during exercise, although they improve the detection of subclinical HF [20, 21].

Building upon this meta-analysis, through the inclusion of new prospective evidence [22–25], and the advancement of the subclinical HF paradigm from cardiac imaging at rest to exercise [20, 21], this review aimed to address the full scope of prospective imaging evidence on the effects of exercise interventions on cardiac structure and function at rest and exercise in patients with T2D. The secondary aim was to address the methodological shortcomings of previous studies and outline the future directions.

## Methods in brief

**Fig. 1:**
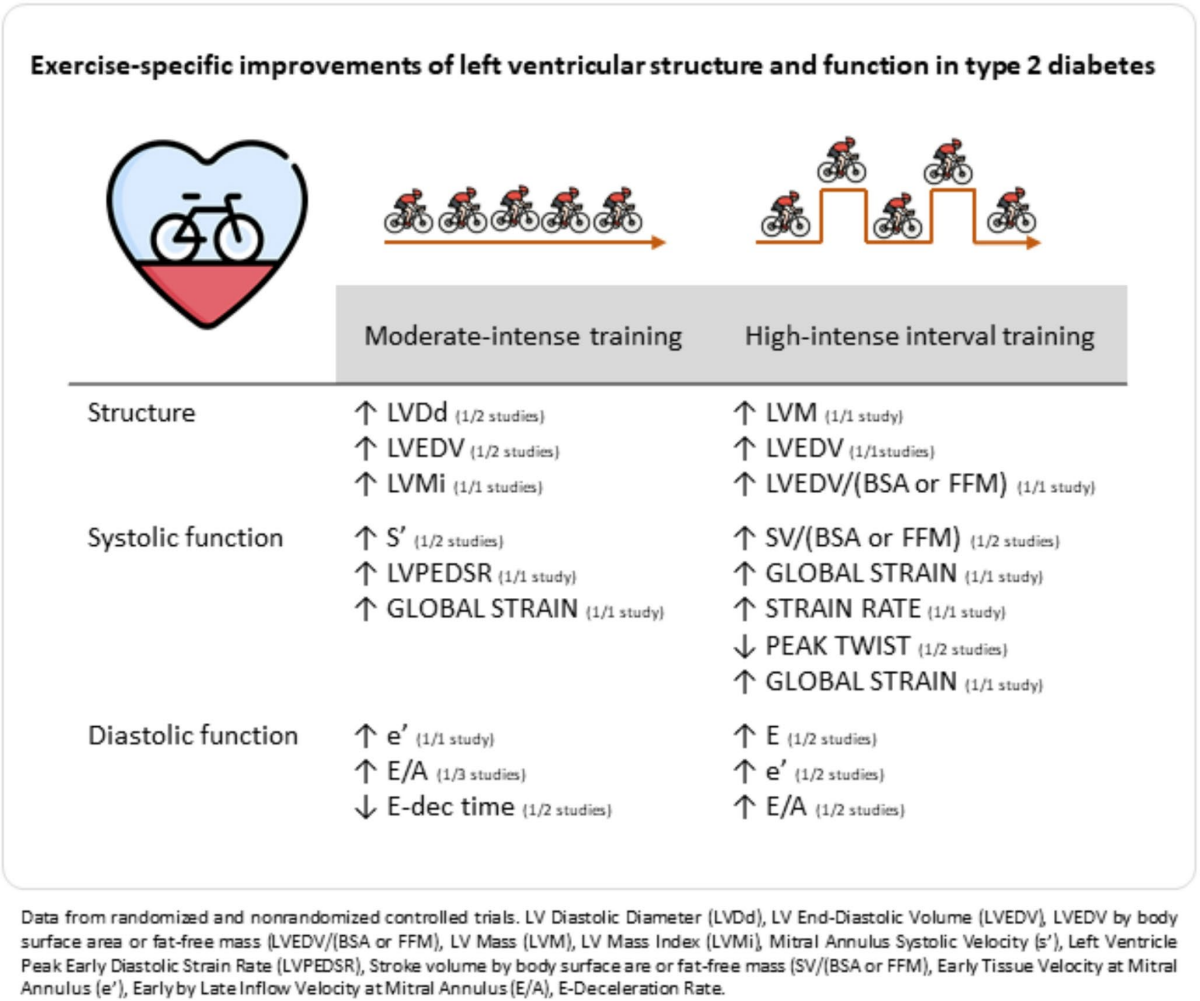
Central illustration– Exercise-specific effects of exercise on cardiac function in patients with type 2 diabetes

We searched two databases (PUBMED and SCOPUS) from inception to 2021 and PUBMED alone in 2024, to find randomized controlled trials (RCT), randomized trials (RT), controlled trials (CT), and single-group (SG) studies in adults with T2D, with exercise interventions of at least four weeks containing cardiac imaging outcomes. Mesh terms were “type 2 diabetes”, “exercise” and “training”, and the query box contained: (((type 2 diabetes) OR (t2d) OR (t2dm) OR (non-insulin-dependent diabetes)) AND ((exercise) OR (training)). The filters were “clinical trials” and “English language” (Fig. 1). We used Rayyan software [26], Zotero reference manager (George Mason University, VG), and spreadsheet Excel 2019 to eliminate duplicates and extract data. We noted between-group differences or group-time interactions (#), within-group changes (↑ or ↓), and time effects (†). Study quality was assessed with the TESTEX scale (Supplement 5) [27]. Tabular data are preintervention values. Supplement 1 contains methods according to the Preferred Reporting Items for Systematic Reviews and Meta-Analyses (PRISMA) Statement.

## Results and discussion

### Study retrieval, populations, and interventions

From the initial 26,882 records, eighteen studies remained, of which two presumably fragmented [28–31] (Supplement 2 - Flowchart). Twelve studies were RCTs, one CT, two RTs and three SG (Table 1). In total, 570 men and women with T2D were enrolled (353 in exercise groups, and 217 in control groups, Table 1). Studies included patients with T2D with and without insulin use, and predominantly no diabetic complications nor known cardiovascular disease (Supplement 3). Patients were overweight or obese (BMI:28–35 kg/m²), middle-aged (age:46-66y), with a poor-to-well-controlled glycemic state (Hba1c: 5.8–8.2%), blood pressure (122-146mmHg) and lipid profile (total cholesterol: 151-205 mg/dl, triglycerides: 97-195 mg/dl, LDL: 89-135 mg/dl) (Supplement 3).

**Table 1:** Baseline characteristics, body composition, metabolic profile and blood pressure of participants in included studies

Study	Design	Group	n	Sample size calculation* (yes/no/unclear)	Age (years)	Sex (M/F)	BMI (kg/m^2^)	Hba1C*(%)*	SBP (mmHg)	DBP (mmHg)	Cholesterol(mg/dl)
McGavock 2004(39)	RCT	CON	11	no	58±7	0/11	34±5	6.6±0.9	133±15	76±9	
		MICT+RT	7		59±5	0/7	34±7	7.4±1.1	139±17	74±11	
Van Ryckeghem 2022(23)	RT	MICT	9	no	66±11	8/1	30±7	6.8±0.5			160±40
		HIIT	10		61±5	9/1	30±5	6.6±0.7			164±19
Brassard 2007(33)	RCT	MICT	11	no	58±5		28±3	5.8±1.3	132±13	76±10	205±43
		CON	12		57±6		31±3	6.4±1.2	142±17	75±11	205±31
Loimaala 2007(40)	RCT	MICT+RT	24	unclear	52±8	24/0		8.2(2.1)	144(17)	88(9)	179(7)
		CON	24		52±8	24/0		8.0(1.3)	146(15)	89(6)	188(7)
Hordern 2014 & Hare 2011(28,31)	RCT	MICT+RT+Diet	88	yes	56±12	46/34	32±5	7.5±1.6	137±17	83±10	189±35
		CON	88		55±9	50/38	32±6	7.6±1.3	129±15	80±9	186±39
Schrauwen-Hinderling 2011(35)	SGT	MICT+RT	11	unclear	60[1]	11/0	30[1]	7.1[0.2]			190[8]
Schmidt 2013(32)	CT	SOCCER	12	no	51±7	12/0	30±3	7.4±1.2	138±15	89±7	170±35
		CON	9		49±9	9/0	30±7	7.5±1.2	126±14	84±8	151±35
Gulsin 2020(22)	RCT	MIT	22	yes			33(32-35)	7.4(1.1)	136 (17)	87(8)	
		CON	30				35(33-41)	7.3(0.9)	138 (13)	85(7)	
Wilson 2019(24)	RCT	HIIT	11	no	52±5	7/4	34±2	7.7±3.1			
		CON	5		51±5	3/2	32±3	7.8±3.3			
Suryanegara 2019(25)	RCT	HIIT	13	yes	61±9	3/10	31±5	7.1±3.1	142±17	89±13	162±43
		CON	13		60±9	3/10	32±5	7.0±2.7	141±14	86±10	167±39
Cassidy 2016(34)	RCT	HIIT	12	unclear	61±9	10/2	31±5	7.0±1	123±4	81±2	155±39
		CON	11		59±9	8/3	32±6	7.0±0.5	126±3	84±2	174±35
Heiskanen 2017(38)	RT	SIT	11	no	49(47,51)	7/4	31(29,33)	5.7(5.4,6.0)	135(129,142)	86(81,90)	
		MICT	10		49(46,51)	6/4	31(29,33)	5.8(5.5,6.0)	146(141,152)	89(85,93)	
Sacre 2014(37)	RCT	MIT+RT+HOME	24	no	59±10	13/11	32±6	7.7±1.6	131(3)	76(1)	179±40
		CON	25		60±9	10/15	32±5	7.7±1.7	122(3)	70(1)	184±37
Hollekim-Strand 2014 & 2016(29,30)	RCT	Home MIT	17	no	55±5	11/6	30±4	6.7±0.7	135±12	81±7	
		HIIT	20		59±5	12/8	30±3	7.0±1.2	142±18	81±7	
Cugusi 2015(36)	SGT	AQUATIC	18	no	52±9	18/0	31±5	8.1±0.8	131±17	83±10	
Jonker 2013(16)	SGT	MIT+RT+Trekking	12	no	46±2	7/5	29±1	6.7±0.3	142±7	87±4	

Data are mean±SD, mean[SEM] or median(IQ) of baseline data. RCT-randomized controlled trial, SGT-single group trial, CON-control, MI(C)T-moderate intense (continuous) training, RT-resistance training, HOME-home training, AQUA-aquatic training, BMI-body mass index, Hba1c-hemoglobin A1C, SBP-systolic blood pressure, DBP-diastolic blood pressure, Cholesterol-total cholesterol, sample size calculation– “yes” if based on the cardiac outcome with the reference provided

Two studies investigated the effects of moderate-intensity exercise training (MIT) or moderate-intensity continuous exercise training (MICT), seven combined moderate-intensity and dynamic resistance exercise training (MIT + RT), five high-intensity interval exercise training (HIIT), one MICT vs. sprint-interval exercise training (SIT), one MICT vs. HIIT, one soccer training and one aquatic training (Table 2). Interventions lasted 2.5–12 months, training frequency was 3-4x/week, endurance exercise intensity between 55 and 100% of the maximal heart rate or maximal oxygen consumption or wattage, and resistance training intensity 55–80% of one maximal repetition or maximal voluntary contraction (Table 2). Eight studies reported supervised exercise training, three semi-supervised exercise training, and five unclear supervision. Training attendance was reported in 6/18 studies and ranged from 60 to 100% (Table 2).

**Table 2:** Intervention description: frequency, intensity, time and type of exercise

Study	Group	Time (months)	Frequency (t/week)	Intensity	Time(min/training)	Type	Adherence(%trainings)	Supervised
McGavock 2004(39)	MICT+RT	≈2.5	3	ET: 65-75%HRR, RT: 50-65%1RM	ET: 30-50RT: 3x10-15rep	ET: cyclingRT: 7 exercises	92±3	/
	CON	≈2.5	/	Usual care	/	/	/	/
Van Ryckeghem 2022(23)	MICT	≈6	3	70-80%HRpeak	35	Cycling	/	yes
	HIIT	≈6	3	Part1: 70-80%HRpeakPart2: 90-100%Wpeak+70%HRpeakPart3: 90-100%Wpeak+70%HRpeakPart4: 90-100%Wpeak+70%Hrpeak	35Phase2: 6x1+6x4Phase3: 7x1+7x4Phase4: 8x1+8x4	Cycling	/	yes
Brassard 2007(33)	MICT	3	3	60-70% VO2peak	30-60	Cycling	/	yes
	CON	3	/	Usual care	/	/	/	/
Loimaala 2007(40)	MICT+RT	12	4ET: 2RT: 2	ET: HR at 65-75%VO2peakRT: 70-80%1RM, 3x12rep	ET: ≤30	ET: walking or joggingRT: 8 exercises for arms, legs and trunk	/	semi
	CON	12	/	usual care	/	/	/	/
Hordern 2014 & Hare 2011(28,31)	MICT+RT+Diet	12	Month 1: 3Months 2-12:/	ET: BORG 12-13/20RT: 3x12-15RM	150min/week	Month 1: gym ET+RTMonth 2-12: HomeET: walking, jogging, cycling, swimmingRT: gym machines, thera-bands, free weights	/	semi
	CON	12	/	Usual care	/	/	/	/
Schrauwen-Hinderling 2011(35)	MICT+RT	≈3m	ET: 2RT: 1	ET: 55%WmaxRT: 1x8rep, 55%MVC2x8rep, 75%MVC	ET: 30	ET: cyclingRT: chest press, leg extension, lat pull-down, leg press, triceps curls, biceps curls, abdominal crunches, horizontal row	/	yes
Schmidt 2013(32)	SOCCER	≈6m	2	Soccer: 43±22% training time >85%HRpeak	5x10=502min breaks	Indoor soccer	60%	yes
	CON	≈6m	/	usual care	/	/	/	/
Gulsin 2020(22)	MIT	≈3m	/	HR at ≈60% VO2peak	≤50	Walking and/or cycling	/	yes

Study	Group	Time (m)	Frequency (n/week)	Intensity	Time(min/training)	Type	Adherence(%trainings)	Supervised
Wilson 2019(24)	HIIT	≈3	3	≥10min at ≥90%HRmaxMonth 1: 1 min intervals, 1 min rest Month 2: 2 min intervals, 2 min restMonth 3: 3 min intervals, 2 min rest	20	/	78±4	/
	CON	≈3	/	usual care	/	/	/	/
Suryanegara 2019(25)	HIIT	≈3	3	Part 1: Borg 9-13/20Part 2: 5x2min, 80rpm, Borg 16-17/20 with 3 min breaks (90s active cycling, 90 s passive). Progress: +10s/week till 3min50s	<40	Cycling	/	no
	CON	≈3	/	usual care	/	/	/	/
Cassidy 2016(34)	HIIT	≈3	3	Part 1: 5 min, Borg 9-13/20Part 2: 5x2min, 80rpm, Borg 16-17/20 with 3 min breaks (90s active cycling, 60 s band-resisted upper body exercise, 30 s passive)Progress: +10s/week to 3min50s	<40	/	100±3	no
	CON	≈3	/	usual care		/	/	/
Heiskanen 2017(38)	SIT	0.5	0-6	4-6x30s all-out cycling at 10% fat-free mass, 4 min rest in-between	<27	Cycling	/	yes
	MICT	0.5	0-6	60%Wmax	40-60	Cycling	/	/
Sacre 2014(37)	MIT+RT+ HOME	6	Gym: 2Home:/	Moderate-vigorous: by RPE and HR	150 min/week ET:20-40RT: 6-12exercises	/	/	semi
	CON	6	/	usual care	/	/	/	/
Hollekim-Strand 2014 & 2016(29,30)	Home MIT (sham)	3	/	/	≥10min/bout; 210min/week	/	94	yes
	HIIT	3	3	Part 1: 10 min at 70%HRmaxPart 2: 4†4min at 90%−95% HRmax 3 min rest between bouts at 70%HRmaxPart 3: 5 min cool-down	404x4min	Walking/jogging (inclined treadmill)	/	yes
Cugusi 2015(36)	AQUATIC	12	3	50-75% VO2max	50	Pool exercises	96	
Jonker 2013(16)	MIT+RT+Trekking	≈7	MIT: 1RT: 2Trekking: 7	/	3.5-4h/weekTrekking= 4-7h/d	MIT:/RT:/Trekking:/	/	/

MICT-moderate-intense continuous training, HIIT-high-intense interval training, RT-resistance training, CON-control group, ET-endurance training, “/”-not reported, Rep-repetitions, HR-heart rate, W-workload, VO2-oxygen consumption, 1RM-one maximal repetition, MVC-maximal voluntary contraction, semi-semisupervised.MICT-moderate-intensity continuous training, HIIT-high-intense interval training, MIT-moderate intense training, RT-resistance training, CON-control group, ET-endurance training, “/”-not reported, Rep-repetitions, HR-heart rate, W-workload, VO2-oxygen consumption, 1RM-one maximal repetition, MVC-maximal voluntary contraction

### Effects of exercise on the left cardiac structure

Table 3 and the central illustration (Fig. 1) qualitatively summarize the effects of exercise on cardiac structure and function in patients with T2D stratified per exercise intervention type. Exercise interventions had mixed between-group effects on the left ventricle diastolic diameter (LVDd) [28, 31–33], left ventricle end-diastolic volume (LVEDV) [22, 32, 34], LVEDV per body surface area or fat-free mass (LVEDV/(BSA or FFM) [22, 24], left ventricle mass and left ventricle mass index (LVM or LVMi) [22, 24, 28, 31–34](Supplement 4: Table 1, and 2), but no between-group effects on diastolic interventricular septal diameter (IVSd) [33], systolic and diastolic left ventricle posterior wall diameter (LVPWs, LVPWd) [33, 34], eccentricity ratio [34], left ventricle end-systolic volume (LVESV) [34], LVESV by body surface area or fat-free mass (LVESV/(BSA or FFM)) [24], and left atrial volume (LAV) [33] (Supplement 4: Table 1, and 2). One single group study showed a reduction in LVESV [35], two no effects on LVESV [16, 36], and three no effects on LVEDV [16, 35, 36].

**Table 3:** Effects of exercise interventions on the cardiac structure and function at rest in type 2 diabetes– summary of controlled trials

Cardiac structure	MIT	HIIT	MIT+RT	Systolic function	MIT	HIIT	MIT+RT	Diastolic function	MIT	HIIT	MIT+RT
IVSd	-	?	?	s’	-	+/-	-	E	-	+/-	-
LVPWd diastolic	-	-	?	LVEF	-	-	-	e’	+	+/-	-
LVPWd systolic	-	-	?	LVSV	?	-	?	E/e’	-	-	-
LVDd	+/-	?	-	LVSV(BSA or FFM)	?	+	?	A	-	-	-
LVSd	-	?	-	LVCO	?	-	?	Early LV filling rate	?	+	?
Eccentricity ratio	?	-	?	LVCO(BSA or FFM)	?	-	?	Late LV filling rate	?	-	?
LVEDV	+/-	+/-	?	Stroke work	?	-	?	E/A	+/-	+/-	-
LVEDV/(BSA or FFM)	-	+/-	?	LVdisplacement	-	?	?	E-deceleration time	+/-	?	-
LVESV	?	-	?	Global strain	-	+/-	-	IVRT	-	?	?
LVESV/(BSA or FFM)	?	-	?	Strain rate	-	+	-	LVPEDSR	+	?	?
LVM	-	+/-	?	Circumferential strain	?	-	?	Perfusion reserve	-	?	?
LVMi	+/-	-	?	Peak twist	?	+/-	?	Aortic distensibility	-	?	?
LAV	-	?	?	LV basal rotation	?	-	?	Pva	?	?	-
Integrated backscatter	?	?	-	LV basal twist rate	?	-	?	Pvs/Pvd	?	?	-
				LV basal untwist rate	?	-	?				
				Time to LV basal untwist	?	-	?				
				LV apical rotation	?	-	?				
				LV apical twist rate	?	-	?				
				LV apical untwist rate	?	-	?				
				Time to LV basal untwist	?	-	?				
				Peak LV twist rate	?	-	?				
				Peak LV untwist rate	?	-	?				
				Time to peak untwist rate	?	-	?				

“+” all CTs favor intervention over control, “+/-“ some CTs do and some do not favor intervention over control, “-“CTs do not favor intervention vs control, “?” - no CT has ever examined intervention vs control, MIT-moderate-intense training, HIIT-high-intense interval training, SIT-sprint interval training, MIT+RT-moderate intense+resistance training; IVSd-interventricular septal diameter diastolic, LVPWd-left ventricle posterior wall diameter, LVDd/s-left ventricle diastolic/systolic diameter, LVEDV-left ventricle end-diastolic volume, LVEDV/(BSA or FFM)-LVEDV per body surface area or fat-free mass, LVESV-left ventricle end-systolic volume, LVESV/(BSA or FFM)-LVESV per body surface area or fat-free mass, LVM-left ventricle mass, LVMi-LVM per body surface area/volume,, LAV-left atrial volume, s’- mitral annulus systolic velocity via pulsed wave (pwTDI) or colored(cTDI) tissue doppler imaging, LVEF-left ventricle ejection fraction, LVSV-left ventricle stroke volume, LVSV/(BSA or FFM - LVSV per body surface area or fat-free mass, LVCO-left ventricle cardiac output, LVCO/(BSA or FFM) - LVCO per body surface area or fat-free mass, LVdis-left ventricle displacement, E and A-early and late inflow velocity at mitral annulus, e’pwTDI or e’colorTDI-early tissue velocity at mitral annulus via pulsed wave or colored tissue doppler imaging, E/e’-early inflow velocity by early tissue velocity at mitral annulus, E/A-early by late inflow velocity at mitral annulus, IVRT-isovolumetric relaxation time, LVPEDSR-left ventricle peak early diastolic strain rate, Pva and Pvs/Pvd -pulmonary venous flow during atrial systole by diastole

### Effects of exercise on the left systolic function

Exercise interventions showed between-group increases in relative left ventricle stroke volume (LVSV/(BSA or FFM)) [24], and mixed effects on increases in the mitral annulus systolic velocity (s’) [24, 28–32, 37], global strain [22, 30, 32, 34, 37, 38], and global strain rate [22, 29–31, 37], and decreases in peak twists [29, 30, 34]. Exercise intervention also showed between-group effects via decreases in the LVEF and stroke work (SW) at iso-intensity during low- and moderate-intense exercise (Supplement 4: Tables 4, 5, 6, 7, 8 and 9) [24]. Single-group studies had mixed effects on s’ [36], LVCO and LVCO/BSA [16, 35](Supplement 4: Table 4, and 5).

Exercise interventions did not show between-group effects in the left ventricle ejection fraction (LVEF) [22, 24, 28, 31–34, 37], cardiac output (CO) [25, 34], CO/(BSA or FFM) [24], LVSV [25, 34], global systolic contraction amplitude (LV displacement) [32], peak endocardial and whole wall circumferential strains [34], peak left ventricular basal and apical rotations [30], twist and untwist rates [30], peak twist rates [30], and times to peak basal, apical, total untwist rates [30] and SW at rest [24] (Supplement 4: Tables 4, 5, 6, 7 and 8). Single-group studies did not affect LVEF [16, 36], LVSV [16], and strain rate [36] (Supplement 4: Tables 4 and 6).

### Effects of exercise on the left diastolic function

Exercise interventions showed between-group effects via increases in the early left ventricular filling rate [34], and left ventricular peak early diastolic strain rate (LVPEDSR) [39] (Supplement 4: Tables 10 & 12). Exercise had mixed between-group effects on the increases in early inflow velocity at mitral annulus (E) [24, 29, 33, 39, 40], early tissue velocity at mitral annulus (e’) [24, 28–32, 37], E/A [22, 24, 29, 30, 32, 33, 37, 39], and decreases in E-deceleration time [32, 33, 37, 39] (Supplement 4: Table 10, and 11). However, exercise did not show between-group effects in E/e’ [22, 24, 29, 32, 37, 40], late inflow velocity at the mitral annulus (A) [24, 33, 39, 40], late left ventricular filling rate [34], isovolumetric relaxation time (IVRT) [33], pulmonary venous flow during atrial systole (Pva) [39], pulmonary venous flow in systole by diastole (Pvs/Pvd) [39], myocardial perfusion reserve [22] and aortic distensibility [22] (Supplement 4: Table 11, and 12). Single-group studies had mixed effects on decreases in E/e’ [32, 37], and no effects on E/A [16] and E-deceleration peak [16] (Supplement 4: Table 10, and 11).

### Effects of exercise on the right cardiac function and fibrosis

One controlled trial showed a between-group effect in tricuspid annular plane systolic excursion (TAPSE) owing to its increase after soccer training (Table 8) [32], and one RCT showed no between-group effect on the integrated backscatter, despite its reduction within the exercise group (Supplement 4: Table 3) [37]. 

### Effects of exercise on the markers of heart failure with preserved ejection fraction

HF with preserved ejection fraction (HFPEF) comprises 75% of all HF diagnoses in T2D [7], and it can be diagnosed invasively and non-invasively. The invasive investigation involves hemodynamic exercise testing for obtaining LV end-diastolic pressure (LVEDP), pulmonary capillary wedge pressure (PCWP) [20, 41] and pulmonary capillary wedge pressure by cardiac output slope (PCWP/CO slope) [41, 42]. The non-invasive imaging includes E, E/A, e’, E/e’, LA volume index (LAVI), and tricuspid regurgitation peak velocity (TRV) at rest [43, 44], and s’, E/e’ and mean pulmonary artery pressure by cardiac output slope (mPAP/COslope) during exercise [45]. The probability scores H2FPEF [46] and HFAPEFF [47] additionally focus on systolic pulmonary artery pressure (sPAP) [46, 47], global longitudinal strain [47], LVMi [47], relative wall thickness [47] and LV wall thickness (LVWd) [47] to estimate the risk of HFPEF. From these HFPEF markers, exercise interventions can affect global strain [18, 32], E [29, 30], e’ [29, 30, 32], E/A [29, 30, 32] and LVMi [34], but not systolic [34] and diastolic posterior LVWd [33, 34] in T2D. The effects of exercise interventions on LAVi, TRV, sPAP, RWT, LVEDP, PCWP, E/e’, s’, PCPW/COslope, and HFPEF probability scores remain unknown.

### Exercise intervention-specific cardiac effects in T2D: intensity and duration

In healthy adults, cardiac adaptations to exercise interventions are intervention- [48] and person-specific [49]. In T2D, only two non-controlled studies compared the effects of different exercise interventions on cardiac variables (Table 3) [23, 38]. In the first study, moderate-intense continuous training (MCIT) was superior to high-intense interval training (HIIT) in increasing left ventricle outflow tract diameter (LVOT), but there were no between-group differences in other parameters of cardiac structure and diastolic function at rest (IVSd, LVPWd, LVDd, s’, IVCT, ET, E, e’, E/e’, A, a’, E/A, E-deceleration time and IVRT), and systolic and diastolic function at peak exercise (LV cardiac output (LVCO), cardiac index (CI), and global strain, E, e’, E/e’ and ejection time (ET)). However, E/e’ did reduce within the HIIT group, and ET increased within the MICT group [23]. 

In the second study, the MICT was superior to sprint interval training (SIT) in increasing LVSV/BSA, the absolute and relative right ventricular mass (RVmass and RVmass/BSA), and right ventricular end-diastolic volumes (RVEDV and RVEDV/BSA), but not right ventricular end-systolic volumes (RVESV and RVESV/BSA) [38] (Supplement 4: Table 3). There were also no between-group differences in the right ventricular systolic function (RVSV, RVSV/BSA, RVCO, RVCO/BSA, RVEF), and left ventricular structure and systolic function (left ventricular work, work index, LVEF, LVSV, LVCO, LVCO/(BSA or FFM), LVESV, LVESV/(BSA or FFM), LVM and LVMi) [38]. Despite this, time effects for the latest six left ventricular variables were primarily driven by changes in the MICT group, which might lead to significant differences on a bigger sample [38]. To conclude, these non-controlled studies indicate that MICT is more effective than SIT, but probably similar to HIIT in improving cardiac structure and function in T2D.

Although RCTs directly comparing exercise groups are lacking, two author groups hypothesized which exercise intensity and duration might induce the greatest diastolic improvements in T2D. Brassard et al. first showed that exercise intervention can improve diastolic strain rate in patients with T2D and mild, but not severe diastolic dysfunction, and hypothesized that exercise interventions longer than three months might maximize cardiac benefits [33]. And secondly, Hordern et al. hypothesized that exercise intensity, and not just the duration [33], might maximize benefits, as T2D patients with the biggest increases in moderate and vigorous physical activity, had the greatest improvements in diastolic cardiac function [28].

### Methodological shortcomings of previous studies

Prospective studies from this review received a modest average TESTEX score of 6.25/15 points for quality of study and reporting (Supplement 4). Studies adequately reported the inclusion criteria, between-group p-values for primary outcomes, and baseline glycemic values, but not randomization, concealment, between-group point estimates, adherence, attendance, and adverse events (Supplement 4).

Moreover, the included studies did not report the inter-rater reliability for the cardiac imaging outcomes, which might limit their clinical usability. Furthermore, only two studies had > 25 participants per group, and only three studies based sample size on cardiac outcomes, thereby risking obtaining false negative findings [50]. Conversely, the absence of corrections for multiple comparisons elevated the risk of false positive findings [51]. 

Next, participants were not included in the studies by following the same diagnostic criteria, although prognosis [52], and possibly cardiac effects, depend on it. The risk of the new onset cardiovascular disease differs > 25% between T2D phenotypes [10], and equalizing the between-group risk at baseline is desirable. The confounders of prognosis are outlined in the ESC’s 2023 risk stratification tool “SCORE2-Diabetes” [10], and include: age, sex, smoking status, geographic region, systolic blood pressure, total and HDL cholesterol, diabetes duration, HbA1c, and kidney function (eGFR). In this review, however, only one prospective study reported kidney function via eGFR [37].

Furthermore, only one study accounted for between-group differences in medication intake [22]. Antidiabetic medications sodium-glucose cotransporter-2 (SGLT2) inhibitors, and glucagon-like peptide-1 receptor agonists (GLP-1 RA), reduce major adverse cardiovascular events in T2D [13, 14], while dipeptidyl peptidase IV (DPP4) inhibitor saxagliptin increases the risk of HF [53]. Finally, dyspnea during daily life might influence exercise-induced cardiac changes, as dyspneic T2D patients have worse cardiac function than non-dyspneic ones [54], and exercise interventions longer than three months might therefore be needed to reverse this dysfunction [33].

Despite the known sex differences in cardiac structure and function in patients with T2D [54] and non-diabetic adults [55], studies did not report sex-specific analyses, exclusive one study in women [39]. Stratification by sex is desirable due to the known sex-specific cardiac changes in aging, pressure and volume overload, and after acute ischemia in healthy adults [56]. Further, changes in cardiac function in athletes seem to be exercise dose-dependent [57]. Yet, two-thirds of the studies did not report exercise adherence (Table 2). Finally, overweight and obese patients with T2D [58] are prone to weight loss after an exercise intervention [11], which can independently affect cardiac function and mask the effects of exercise [15, 59–61]. 

### Future directions

The effects of exercise interventions on HFPEF markers LAVi, TRpV, sPAP, relative wall thickness, PCWP^48^, PCWP/CO, and left and right atrial function inclusive right ventricular structure and function are unknown. Secondly, only one study examined cardiac changes at peak exercise [23], but cardiac imaging during exercise improves the detection of HFPEF [20, 21] and could be more sensitive than imaging at rest in capturing cardiac improvements. As outlined herein and previously [17], an absence of controlled trials with multiple exercise groups precludes knowing which exercise frequency, intensity, duration and volume improve cardiac structure and function the most in T2D. Finally, it is unknown whether cardiac changes translate to a better prognosis.

## Conclusion

Exercise interventions can improve left ventricular structure and function at rest and during exercise and right ventricular systolic function at rest in patients with T2D. Future studies should investigate sex- and phenotype-specific effects of exercise on the right heart during rest and peak exercise, determine the optimal exercise intensity, duration and volume for inducing cardiac changes, and the translation of cardiac changes into long-term prognosis.

## Electronic supplementary material

Below is the link to the electronic supplementary material.

Supplementary Material 1

Supplementary Material 2

Supplementary Material 3

## Data Availability

No datasets were generated or analysed during the current study.

## Abbreviations

**Cardiac structure**: diastolic interventricular septal diameter (IVSd), systolic and diastolic left ventricle posterior wall diameter (LVPWs, LVPWd), left ventricle diastolic diameter (LVDd), left ventricle systolic diameter (LVSd), eccentricity ratio, left ventricle end-diastolic volume (LVEDV), left ventricle end-diastolic volume per body surface area or fat-free mass (LVEDV/(BSA or FFM), left ventricle end-systolic volume (LVESV), left ventricle end-systolic volume per body surface area or fat-free mass (LVESV/(BSA or FFM), left ventricle mass (LVM), left ventricle mass per body surface area or volume (LVMi), left ventricle outflow tract diameter (LVOT), left atrial volume (LAV), right ventricle mass (RVmass), right ventricle mass per body surface area of fat-free mass (RVmass/BSA), right ventricle end-systolic volume (RVEDV), right ventricle end-systolic volume (RVESV), right ventricle end-systolic volume per body surface area (RVESV/BSA), and calibrated integrated backscatter.
**Systolic function**: tricuspid annulus systolic velocity via pulsed wave (pwTDI) or colored(cTDI) tissue doppler imaging (s’), isovolumetric contraction time (IVCT), ejection time (ET), left ventricle ejection fraction (LVEF), left ventricle stroke volume (LVSV), left ventricle stroke volume per body surface area or fat-free mass (LVSV/(BSA or FFM)), left ventricle cardiac output (LVCO), left ventricle cardiac output per body surface area or fat-free mass (LVCO/(BSA or FFM)), stroke work (SW), left ventricle displacement (LVdis), right ventricle stroke volume (RVSV), right ventricle stroke volume per body surface area (RVSV/BSA), right ventricle cardiac output (RVCO), right ventricle ejection fraction (RVEF), tricuspid annular plane systolic excursion (TAPSE), global strain, strain rate, peak endocardial circumferential strain, peak whole wall circumferential strain, peak twist, peak LV basal rotation, LV basal twist rate, time to peak basal untwist rate, peak LV apical rotation, LV apical twist rate, LV apical untwist rate, time to LV apical untwist rate, peak LV twist rate, peak LV untwist rate, time to peak untwist rate, and cardiac index (CI).
**Diastolic function**: early inflow velocity at mitral annulus (E), early tissue velocity at mitral annulus via pulsed wave tissue Doppler imaging (e’pwTDI), early tissue velocity at mitral annulus via colored tissue Doppler imaging(e’ colorTDI), early inflow velocity at mitral annulus by early tissue velocity at mitral annulus(E/e’), late inflow velocity at mitral annulus (A), late tissue velocity at mitral annulus (a’), late LV filling rate, early by late inflow velocity at mitral annulus (E/A), peak E-deceleration, E-deceleration, isovolumetric relaxation time (IVRT), left ventricle peak early diastolic strain rate (LVPEDSR), myocardial perfusion reserve, aortic distensibility, pulmonary venous flow during atrial systole (Pva), and pulmonary venous flow in systole by pulmonary venous flow in diastole (Pvs/Pvd).

## Declarations

### Additional materials

Supplements 1–3. Methods, flowchart, lipid profile, and inclusion criteria of individual studies.

Supplement 4. Tables 1–13: The effects of exercise on cardiac structure and function in T2D.

Supplement 5. Study quality assessment.

## References

[1] Rubler S, Dlugash J, Yuceoglu YZ, Kumral T, Branwood AW, Grishman A (1972) New type of cardiomyopathy associated with diabetic glomerulosclerosis. Am J Cardiol 30(6):595–602 4263660 10.1016/0002-9149(72)90595-4

[2] Athithan L, Gulsin GS, McCann GP, Levelt E (2019) Diabetic cardiomyopathy: pathophysiology, theories and evidence to date. World J Diabetes 10(10):490–510 31641426 10.4239/wjd.v10.i10.490 PMC6801309

[3] Jia G, Hill MA, Sowers JR (2018) Diabetic cardiomyopathy. Circ Res 122(4):624–638 29449364 10.1161/CIRCRESAHA.117.311586 PMC5819359

[4] Marso SP, Bain SC, Consoli A, Eliaschewitz FG, Jódar E, Leiter LA et al (2016) Semaglutide and cardiovascular outcomes in patients with type 2 diabetes. N Engl J Med 375(19):1834–1844 27633186 10.1056/NEJMoa1607141

[5] Zinman B, Wanner C, Lachin JM, Fitchett D, Bluhmki E, Hantel S et al (2015) Empagliflozin, cardiovascular outcomes, and mortality in type 2 diabetes. N Engl J Med 373(22):2117–2128 26378978 10.1056/NEJMoa1504720

[6] White WB, Cannon CP, Heller SR, Nissen SE, Bergenstal RM, Bakris GL et al (2013) Alogliptin after acute coronary syndrome in patients with type 2 diabetes. N Engl J Med 369(14):1327–1335 23992602 10.1056/NEJMoa1305889

[7] Castagno D, Baird-Gunning J, Jhund PS, Biondi-Zoccai G, MacDonald MR, Petrie MC et al (2011) Intensive glycemic control has no impact on the risk of heart failure in type 2 diabetic patients: evidence from a 37,229 patient meta-analysis. Am Heart J 162(5):938–948e2 22093212 10.1016/j.ahj.2011.07.030

[8] MacDonald MR, Petrie MC, Varyani F, Ostergren J, Michelson EL, Young JB et al (2008) Impact of diabetes on outcomes in patients with low and preserved ejection fraction heart failure: an analysis of the Candesartan in heart failure: assessment of reduction in mortality and morbidity (CHARM) programme. Eur Heart J 29(11):1377–1385 18413309 10.1093/eurheartj/ehn153

[9] Yusuf S, Pfeffer MA, Swedberg K, Granger CB, Held P, McMurray JJ et al (2003) Effects of Candesartan in patients with chronic heart failure and preserved left-ventricular ejection fraction: the CHARM-Preserved trial. Lancet 362(9386):777–781 13678871 10.1016/S0140-6736(03)14285-7

[10] Federici M, Schütt K, Müller-Wieland D, Ajjan RA, Christodorescu RM, Crawford C et al 2023 ESC guidelines for the management of cardiovascular disease in patients with diabetes 10.1093/eurheartj/ehad881 38195096

[11] Colberg SR, Sigal RJ, Yardley JE, Riddell MC, Dunstan DW, Dempsey PC et al (2016) Physical activity/exercise and diabetes: A position statement of the American diabetes association. Diabetes Care 39(11):2065–2079 27926890 10.2337/dc16-1728 PMC6908414

[12] Kanaley JA, Colberg SR, Corcoran MH, Malin SK, Rodriguez NR, Crespo CJ et al (2022) Exercise/Physical activity in individuals with type 2 diabetes: A consensus statement from the American college of sports medicine. Med Sci Sports Exerc 54(2):353 35029593 10.1249/MSS.0000000000002800 PMC8802999

[13] Sattar N, Lee MMY, Kristensen SL, Branch KRH, Prato SD, Khurmi NS et al (2021) Cardiovascular, mortality, and kidney outcomes with GLP-1 receptor agonists in patients with type 2 diabetes: a systematic review and meta-analysis of randomised trials. Lancet Diabetes Endocrinol 9(10):653–662 34425083 10.1016/S2213-8587(21)00203-5

[14] McGuire DK, Shih WJ, Cosentino F, Charbonnel B, Cherney DZI, Dagogo-Jack S et al (2021) Association of SGLT2 inhibitors with cardiovascular and kidney outcomes in patients with type 2 diabetes: A Meta-analysis. JAMA Cardiol 6(2):148–158 33031522 10.1001/jamacardio.2020.4511 PMC7542529

[15] Willens HJ, Chakko SC, Byers P, Chirinos JA, Labrador E, Castrillon JC et al (2005) Effects of weight loss after gastric bypass on right and left ventricular function assessed by tissue doppler imaging. Am J Cardiol 95(12):1521–1524 15950589 10.1016/j.amjcard.2005.02.029

[16] Jonker JT, Snel M, Hammer S, Jazet IM, van der Meer RW, Pijl H et al (2014) Sustained cardiac remodeling after a short-term very low calorie diet in type 2 diabetes mellitus patients. Int J Cardiovasc Imaging 30(1):121–127 24129410 10.1007/s10554-013-0302-y

[17] Verboven M, Van Ryckeghem L, Belkhouribchia J, Dendale P, Eijnde BO, Hansen D et al (2019) Effect of exercise intervention on cardiac function in type 2 diabetes mellitus: A systematic review. Sports Med 49(2):255–268 30357657 10.1007/s40279-018-1003-4

[18] Anand V, Garg S, Garg J, Bano S, Pritzker M (2018) Impact of exercise training on cardiac function among patients with type 2 diabetes: A SYSTEMATIC REVIEW AND META-ANALYSIS. J Cardiopulm Rehabil Prev 38(6):358–365 30142130 10.1097/HCR.0000000000000353

[19] Kang Y, Wang S, Huang J, Cai L, Keller BB (2019) Right ventricular dysfunction and remodeling in diabetic cardiomyopathy. Am J Physiol-Heart Circ Physiol 316(1):H113–H122 30412438 10.1152/ajpheart.00440.2018

[20] McDonagh TA, Metra M, Adamo M, Gardner RS, Baumbach A, Böhm M et al (2021) 2021 ESC guidelines for the diagnosis and treatment of acute and chronic heart failure. Eur Heart J 42(36):3599–3726 34447992 10.1093/eurheartj/ehab368

[21] Saito Y, Obokata M, Harada T, Kagami K, Wada N, Okumura Y et al (2023) Prognostic benefit of early diagnosis with exercise stress testing in heart failure with preserved ejection fraction. Eur J Prev Cardiol 30(9):902–911 37094815 10.1093/eurjpc/zwad127

[22] Gulsin GS, Swarbrick DJ, Athithan L, Brady EM, Henson J, Baldry E et al (2020) Effects of Low-Energy diet or exercise on cardiovascular function in Working-Age adults with type 2 diabetes: A prospective, randomized, Open-Label, blinded end point trial. Diabetes Care 43(6):1300–1310 32220917 10.2337/dc20-0129

[23] Van Ryckeghem L, Keytsman C, De Brandt J, Verboven K, Verbaanderd E, Marinus N et al (2022) Impact of continuous vs. interval training on oxygen extraction and cardiac function during exercise in type 2 diabetes mellitus. Eur J Appl Physiol 122(4):875–887 35038022 10.1007/s00421-022-04884-9

[24] Wilson GA, Wilkins GT, Cotter JD, Lamberts RR, Lal S, Baldi JC (2019) HIIT improves left ventricular exercise response in adults with type 2 diabetes. Med Sci Sports Exerc 51(6):1099–1105 30640284 10.1249/MSS.0000000000001897

[25] Suryanegara J, Cassidy S, Ninkovic V, Popovic D, Grbovic M, Okwose N et al (2019) High intensity interval training protects the heart during increased metabolic demand in patients with type 2 diabetes: a randomised controlled trial. Acta Diabetol 56(3):321–329 30387015 10.1007/s00592-018-1245-5 PMC6394729

[26] Ouzzani M, Hammady H, Fedorowicz Z, Elmagarmid A (2016) Rayyan—a web and mobile app for systematic reviews. Syst Rev 5(1):210 27919275 10.1186/s13643-016-0384-4 PMC5139140

[27] Smart NA, Waldron M, Ismail H, Giallauria F, Vigorito C, Cornelissen V et al (2015) Validation of a new tool for the assessment of study quality and reporting in exercise training studies: TESTEX. JBI Evid Implement 13(1):9 10.1097/XEB.0000000000000020 25734864

[28] Hordern MD, Coombes JS, Cooney LM, Jeffriess L, Prins JB, Marwick TH (2009) Effects of exercise intervention on myocardial function in type 2 diabetes. Heart 95(16):1343–1349 19429570 10.1136/hrt.2009.165571

[29] Hollekim-Strand SM, Bjørgaas MR, Albrektsen G, Tjønna AE, Wisløff U, Ingul CB (2014) High-Intensity interval exercise effectively improves cardiac function in patients with type 2 diabetes mellitus and diastolic dysfunction. J Am Coll Cardiol 64(16):1758–1760 25323267 10.1016/j.jacc.2014.07.971

[30] Hollekim-Strand SM, Høydahl SF, Follestad T, Dalen H, Bjørgaas MR, Wisløff U et al (2016) Exercise training normalizes timing of left ventricular untwist rate, but not peak untwist rate, in individuals with type 2 diabetes and diastolic dysfunction: A pilot study. J Am Soc Echocardiogr 29(5):421–430e2 26948543 10.1016/j.echo.2016.01.005

[31] Hare JL, Hordern MD, Leano R, Stanton T, Prins JB, Marwick TH (2011) Application of an exercise intervention on the evolution of diastolic dysfunction in patients with diabetes mellitus: efficacy and effectiveness. Circ Heart Fail 4(4):441–449 21576281 10.1161/CIRCHEARTFAILURE.110.959312

[32] Schmidt JF, Andersen TR, Horton J, Brix J, Tarnow L, Krustrup P et al (2013) Soccer training improves cardiac function in men with type 2 diabetes. Med Sci Sports Exerc 45(12):2223–2233 23669882 10.1249/MSS.0b013e31829ab43c

[33] Brassard P, Legault S, Garneau C, Bogaty P, Dumesnil JG, Poirier P (2007) Normalization of diastolic dysfunction in type 2 diabetics after exercise training. Med Sci Sports Exerc 39(11):1896–1901 17986895 10.1249/mss.0b013e318145b642

[34] Cassidy S, Thoma C, Hallsworth K, Parikh J, Hollingsworth KG, Taylor R et al (2016) High intensity intermittent exercise improves cardiac structure and function and reduces liver fat in patients with type 2 diabetes: a randomised controlled trial. Diabetologia 59(1):56–66 26350611 10.1007/s00125-015-3741-2 PMC4670457

[35] Schrauwen-Hinderling VB, Meex RC, Hesselink MK, Van De Weijer T, Leiner T, Schär M et al (2011) Cardiac lipid content is unresponsive to a physical activity training intervention in type 2 diabetic patients, despite improved ejection fraction. Cardiovasc Diabetol 10(1):47 21615922 10.1186/1475-2840-10-47 PMC3127755

[36] Cugusi L, Cadeddu C, Nocco S, Orrù F, Bandino S, Deidda M et al (2015) Effects of an Aquatic-Based exercise program to improve cardiometabolic profile, quality of life, and physical activity levels in men with type 2. 7(2):141–148 Diabetes Mellitus. PM&R 10.1016/j.pmrj.2014.09.004 25217820

[37] Sacre JW, Jellis CL, Jenkins C, Haluska BA, Baumert M, Coombes JS et al (2014) A six-month exercise intervention in subclinical diabetic heart disease: effects on exercise capacity, autonomic and myocardial function. Metabolism 63(9):1104–1114 24997499 10.1016/j.metabol.2014.05.007

[38] Heiskanen MA, Sjöros TJ, Heinonen IHA, Löyttyniemi E, Koivumäki M, Motiani KK et al (2017) Sprint interval training decreases left-ventricular glucose uptake compared to moderate-intensity continuous training in subjects with type 2 diabetes or prediabetes. Sci Rep 7(1):10531 28874821 10.1038/s41598-017-10931-9 PMC5585392

[39] McGavock J, Sandra Mandic R, Lewanczuk M, Koller IV, Muhll A, Quinney et al (2004) Cardiovascular adaptations to exercise training in postmenopausal women with type 2 diabetes mellitus. Cardiovasc Diabetol 22(11):685–686 10.1186/1475-2840-3-3 PMC400749 15023235

[40] Loimaala A, Groundstroem K, Rinne M, Nenonen A, Huhtala H, Vuori I (2007) Exercise training does not improve myocardial diastolic tissue velocities in type 2 diabetes. Cardiovasc Ultrasound 5(1):32 17897465 10.1186/1476-7120-5-32 PMC2094704

[41] Ho JE, Zern EK, Wooster L, Bailey CS, Cunningham T, Eisman AS et al (2019) Differential clinical profiles, exercise responses, and outcomes associated with existing HFpEF definitions. Circulation 140(5):353–365 31132875 10.1161/CIRCULATIONAHA.118.039136 PMC6684250

[42] Eisman AS, Shah RV, Dhakal BP, Pappagianopoulos PP, Wooster L, Bailey C et al (2018) Pulmonary capillary wedge pressure patterns during exercise predict exercise capacity and incident heart failure. Circ Heart Fail 11(5):e004750 29695381 10.1161/CIRCHEARTFAILURE.117.004750 PMC5937988

[43] Kossaify A, Nasr M (2019) Diastolic dysfunction and the new recommendations for echocardiographic assessment of left ventricular diastolic function: summary of guidelines and novelties in diagnosis and grading. J Diagn Med Sonogr 35(4):317–325

[44] Nagueh SF, Smiseth OA, Appleton CP, Byrd BF, Dokainish H, Edvardsen T et al (2016) Recommendations for the evaluation of left ventricular diastolic function by echocardiography: an update from the American society of echocardiography and the European association of cardiovascular imaging. J Am Soc Echocardiogr 29(4):277–314 27037982 10.1016/j.echo.2016.01.011

[45] Verwerft J, Verbrugge FH, Claessen G, Herbots L, Dendale P, Gevaert AB (2022) Exercise systolic reserve and exercise pulmonary hypertension improve diagnosis of heart failure with preserved ejection fraction. Front Cardiovasc Med 9:814601 35224049 10.3389/fcvm.2022.814601 PMC8863971

[46] Reddy YNV, Carter RE, Obokata M, Redfield MM, Borlaug BA (2018) A simple, Evidence-Based approach to help guide diagnosis of heart failure with preserved ejection fraction. Circulation 138(9):861–870 29792299 10.1161/CIRCULATIONAHA.118.034646 PMC6202181

[47] Pieske B, Tschöpe C, De Boer RA, Fraser AG, Anker SD, Donal E et al (2019) How to diagnose heart failure with preserved ejection fraction: the HFA–PEFF diagnostic algorithm: a consensus recommendation from the heart failure association (HFA) of the European society of cardiology (ESC). Eur Heart J 40(40):3297–3317 31504452 10.1093/eurheartj/ehz641

[48] Pluim B, Zwinderman A, van der Laarse A, Wall E (2000) The athlete s heart: A Meta-Analysis of cardiac structure and function. Circulation 101:336–344 10645932 10.1161/01.cir.101.3.336

[49] Marsh CE, Thomas HJ, Naylor LH, Dembo LG, Scurrah KJ, Green DJ (2022) Left ventricular adaptation to exercise training via magnetic resonance imaging: studies of twin responses to understand exercise therapy. Med Sci Sports Exerc 54(7):1095–1104 35220371 10.1249/MSS.0000000000002899

[50] Ekkekakis P, Swinton P, Tiller NB Extraordinary Claims in the Literature on High-Intensity Interval Training (HIIT): I. Bonafide Scientific Revolution or a Looming Crisis of Replication and Credibility? Sports Med [Internet]. 2023 Aug 10 [cited 2023 Aug 30]; Available from: https://link.springer.com/10.1007/s40279-023-01880-7 10.1007/s40279-023-01880-7 37561389

[51] Albers C (2019) The problem with unadjusted multiple and sequential statistical testing. Nat Commun 10(1):1921 31015469 10.1038/s41467-019-09941-0 PMC6478696

[52] Magkos F, Reeds DN, Mittendorfer B (2023) Evolution of the diagnostic value of the sugar of the blood: hitting the sweet spot to identify alterations in glucose dynamics. Physiol Rev 103(1):7–30 35635320 10.1152/physrev.00015.2022 PMC9576168

[53] Scirica BM, Bhatt DL, Braunwald E, Steg PG, Davidson J, Hirshberg B et al (2013) Saxagliptin and cardiovascular outcomes in patients with type 2 diabetes mellitus. N Engl J Med 369(14):1317–1326 23992601 10.1056/NEJMoa1307684

[54] Gojevic T, Van Ryckeghem L, Jogani S, Frederix I, Bakelants E, Petit T et al (2023) Pulmonary hypertension during exercise underlies unexplained exertional dyspnoea in patients with type 2 diabetes. Eur J Prev Cardiol 30(1):37–45 35881689 10.1093/eurjpc/zwac153

[55] St. Pierre SR, Peirlinck M, Kuhl E (2022) Sex matters: A comprehensive comparison of female and male hearts. Front Physiol 13:831179 35392369 10.3389/fphys.2022.831179 PMC8980481

[56] Piro M, Della Bona R, Abbate A, Biasucci LM, Crea F (2010) Sex-Related differences in myocardial remodeling. J Am Coll Cardiol 55(11):1057–1065 20223363 10.1016/j.jacc.2009.09.065

[57] Maron BJ, Pelliccia A (2006) The heart of trained athletes: cardiac remodeling and the risks of sports, including sudden death. Circulation 114(15):1633–1644 17030703 10.1161/CIRCULATIONAHA.106.613562

[58] Centers for Disease Control and Prevention (CDC) (2004) Prevalence of overweight and obesity among adults with diagnosed diabetes–United states, 1988–1994 and 1999–2002. MMWR Morb Mortal Wkly Rep 53(45):1066–1068 15549021

[59] Karason K, Wallentin I, Larsson B, Sjöström L (1998) Effects of obesity and weight loss on cardiac function and valvular performance. Obes Res 6(6):422–429 9845232 10.1002/j.1550-8528.1998.tb00374.x

[60] Syed M, Rosati C, Torosoff MT, El-Hajjar M, Feustel P, Alger S et al (2009) The impact of weight loss on cardiac structure and function in obese patients. Obes Surg 19(1):36–40 18780132 10.1007/s11695-008-9645-1

[61] Sargsyan N, Chen JY, Aggarwal R, Fadel MG, Fehervari M, Ashrafian H (2023) The effects of bariatric surgery on cardiac function: a systematic review and meta-analysis. Int J Obes 1–11 10.1038/s41366-023-01412-3 PMC10824663 38007595

