# Supplementary material for: Effects of exercise on cardiac structure and function in patients with type 2 diabetes: a narrative review of prospective imaging studies
Source: Int J Cardiovasc Imaging. 2025 Jul 19;41(10):1851–64. doi: 10.1007/s10554-025-03457-z (PMC12491076; doi:10.1007/s10554-025-03457-z)
Supplement: Supplementary file 1 — Supplementary Material 1 [file 10554_2025_3457_MOESM1_ESM.docx]

# Supplement 1. Methods

## Inclusion criteria

This narrative review was drafted by following The Preferred Reporting Items for Systematic Reviews and Meta-Analysis (PRISMA) checklist. We included peer-reviewed prospective randomized controlled trials (RCT), randomized trials (RT), controlled trials (CT), and single-group (SG) studies in adults, with exercise interventions of at least four weeks, and cardiac imaging outcomes. Studies without cardiac imaging, on patients with pre-diabetes and metabolic syndrome, patients with combined T2D and other diseases, and with dietary or non-exercise interventions, were excluded.

## Sources, search strategy, and selection process

One investigator (TG) searched two databases (Pubmed from inception to 21.02.2024; and Scopus from inception to 2021) to identify relevant articles. Maximally broad search was done with Mesh terms: “type 2 diabetes”, “exercise” and “training”. The query box included: (((type 2 diabetes) OR (t2d) OR (t2dm) OR (non-insulin-dependent diabetes)) AND ((exercise) OR (training)). The filters were set to “clinical trials” and “English language” (Supplement 2. Flowchart). The search records were imported in the online software Rayyan^1^ where duplicates were automatically detected, inspected and removed. The abstracts were manually screened(TG) and eligible studies exported to the reference manager Zotero for the full-text screening (George Maison University, Virginia, USA). One investigator extracted data from included studies into a spreadsheet (Microsoft Excel 2019, Redmond, WA, USA).

## Outcomes

Synthesis contained all imaging data at rest and exercise describing cardiac structure (21 variables), and systolic and diastolic function (34 and 24 variables). Study and population characteristics are in Supplement 3. (Tables 1 and 2). Tabular data are pre-intervention values expressed as originally shown (mean±SD, mean[SEM] or median(IQ)), and graphical data are noted as “graph.” Due to low sample sizes in most studies (<25 patients per group), three types of treatment effects were of interest: 1) between-group differences or group-time interactions (#), 2) within-group changes (↑ or ↓), and 3) time effects (i.e within total sample changes (†). The quality of the study and reporting was graded by one investigator via the TESTEX scale.

**Supplement 2 - Flowchart**

Duplicates: (n = 9721)

Records identified from:

Pubmed + Scopus (inception to 2021, n = 26144)

Pubmed (2021 to 31.05.2023, n = 610)

Pubmed (31.05.2023 to 21.02.2024 n = 128)

**Identification**

Records (abstracts) screened

(n = 17161)

Records (abstracts) excluded

(n = 16235)

Reports sought for retrieval

(n = 926)

Reports not retrieved

(n = 0)

**Screening**

Reports excluded (n = 908)

- animal studies
- no imaging outcomes on humans
- diseases other than type 2 diabetes
- inappropriate or too short intervention

Full text reports screened

(n = 926)

Reports included in the review

(n = 18)

**Included**

*From:*  Page MJ, McKenzie JE, Bossuyt PM, Boutron I, Hoffmann TC, Mulrow CD, et al. The PRISMA 2020 statement: an updated guideline for reporting systematic reviews. BMJ 2021;372:n71. doi: 10.1136/bmj.n71

**Supplement 3. Lipid profile and inclusion criteria of the individual studies**

Table 1. Lipid profile of participants in included studies

| Study | Group  *(n)* | HDL (mg/dl) | LDL (mg/dl) | TRG (mg/dl) |
| --- | --- | --- | --- | --- |
| McGavock 2004^2^ | CON | 50±12 | 112±35 |  |
|  | MICT+RT | 50±8 | 116±23 |  |
| Van Ryckeghem 2022^3^ | MICT | 53±18 | 106±35 | 126±74 |
|  | HIIT | 47±13 | 118±20 | 142±72 |
| Brassard 2007^4^ | MICT | 39±8 | 135±39 | 151±62 |
|  | CON | 39±12 | 128±35 | 195±124 |
| Loimaala 2007^5^ | MICT+RT | 42(2) | 122(7) | 151(13) |
|  | CON | 44(2) | 125(7) | 162(13) |
| Hordern 2014 & Hare 2011^6,7^ | MICT+RT+Diet | 53±13 | 108±31 | 159±80 |
|  | CON | 54±17 | 104±35 | 160±106 |
| Schrauwen-Hinderling 2011^8^ | MICT+RT | 45[4] | 104[7] | 186[18] |
| Schmidt 2013^9^ | SOCCER | 46±8 | 104±35 | 115±44 |
|  | CON | 43±12 | 89±35 | 106±35 |
| Gulsin 2020^10^ | MIT |  |  |  |
|  | CON |  |  |  |
| Wilson 2019^11^ | HIIT |  |  |  |
|  | CON |  |  |  |
| Suryanegara 2019^12^ | HIIT |  |  | 97±27 |
|  | CON |  |  | 97±35 |
| Cassidy 2016^13^ | HIIT |  |  |  |
|  | CON |  |  |  |
| Heiskanen 2017^14^ | SIT |  |  |  |
|  | MICT |  |  |  |
| Sacre 2014^15^ | MIT+RT+HOME | 40±10 | 110±40 | 106(80-163) |
|  | CON. | 44±12 | 109±33 | 133(111-168) |
| Hollekim-Strand 2014 & 2016^16,17^ | Home MIT |  |  |  |
|  | HIIT |  |  |  |
| Cugusi 2015^18^ | AQUATIC |  |  |  |
| Jonker 2013^19^ | MIT+RT+Trekking |  |  |  |

Data are mean±SD, mean[SEM] or median(IQ) of baseline data. CON-control, MI(C)T-moderate intense (continuous) training, RT-resistance training, HOME-home training, AQUA-aquatic training, HDL-high density lipoprotein, LDL-low density lipoprotein, cholesterol-total cholesterol, TRG-triglycerides

Table 2. Inclusion criteria of participants in included studies

| Study | Sample description |
| --- | --- |
| McGavock 2004^2^ | T2D, postmenopausal women, no diabetic complications, ischemic heart disease, angina or cardiac symptoms limiting exercise, musculoskeletal and peripheral vascular abnormalities |
| Van Ryckeghem 2022^3^ | T2D, asymptomatic (no dyspnoea or chest pain at rest or exercise), on stable pharmacological treatment (3m), no renal disease, retinopathy, neurological, orthopedic, oncologic, pulmonary or cardiovascular diseases (e.g. valve disease, coronary artery disease, congenital heart disease) |
| Brassard 2007^4^ | T2D, no insulin and complications (renal failure with creatinine above normal), macroalbuminuria, proliferative retinopathy, sensitive, motor or autonomic neuropathies) |
| Loimaala 2007^5^ | T2D, no insulin, asymptomatic, no myocardial infarction, arrhythmias, ischemia, wall motion abnormalities on resting echocardiogram, valvular disease, lung or connective tissue disease, cardiomyopathy, rheumatoid arthritis or any chronic disease besides T2D |
| Hordern 2014 & Hare 2011^6,7^ | T2D, no CAD, CVD and pregnancy |
| Schrauwen-Hinderling 2011^8^ | T2D, no insulin and cardiac disease, with T2D diagnosis≥1y, baseline plasma glucose≥126mg/dl, SBP≤160mmHg and DBP≤100mmHg |
| Schmidt 2013^9^ | T2D, with and without insulin, stable HbA1C levels, without history or symptoms of CVD, cancer, complications (nephropathy, retinopathy and neuropathy), T1D, beta-blockers or musculoskeletal complaints limiting training |
| Gulsin 2020^10^ | T2D, no insulin, 18-65years, diagnosis between 3m and 12 years ago, BMI>30 kg/m2 (or 27 kg/m2 if South Asian or black ethnicity), ≤3glucose-lowering medications, no history, signs, or symptoms of CVD (including CAD, stroke, transient ischemic attack, peripheral artery disease, or heart failure), no weight loss >5 kg in the preceding 6 months, no contraindication to exercise |
| Wilson 2019^11^ | T2D, with and without insulin, 34-62 years, no CVD, respiratory or microvascular disease, no complications, with negative exercise stress ECG |
| Suryanegara 2019^12^ | T2D, controlled by diet or medication ≥6 months, no complications, smoking, contraindications or inability to exercise, no CAD, medication affecting cardiorespiratory function, eligible if not doing moderate to vigorous exercise. |
| Cassidy 2016^13^ | T2D, stable with diet and/or metformin for ≥6m, no overt cardiac disease, no regular exercise (≥60 min moderate-vigorous physical activity per week), use of beta-blocker, or contraindications to exercise |
| Heiskanen 2017^14^ | T2D + preT2D, 40-55years, BMI 18.5-35 kg/m2, blood pressure ≤160/100 mmHg, no regular exercise (peak oxygen uptake VO2peak≤ 40 ml/kg/min), with defective glucose tolerance according to the criteria of the American Diabetes Association and HbA1c <6.3%. |
| Sacre 2014^15^ | T2D, with and without insulin, age≥40 years, asymptomatic, with a preserved ejection fraction of the left ventricle and subclinical diastolic dysfunction by echocardiography (>1SD below the age-based normal septal early diastolic tissue velocity), without CVD, psychiatric, vascular or other severe diseases, symptomatic macro- or micro-vascular complications or ischemia |
| Hollekim-Strand 2014 & 2016^16,17^ | T2D, no insulin, age 20-65 years, T2D diagnosis ≤10 years, no diastolic dysfunction (e' < 8 cm/s), no overt CVD, atrial fibrillation or other significant cardiac arrhythmias, untreated hypertension, diabetic retinopathy or neuropathy, albuminuria, LVEF<40%, BMI>35 kg/m2, ischemia (exercise echocardiography), pregnancy, inability to exercise, no drug or alcohol abuse, and physical activity level above minimal suggested for T2D |
| Cugusi 2015^18^ | T2D, 40-65 years, LVEF≥55%, no echocardiographic wall motion abnormalities, no hepatic and renal dysfunction (bilirubin≤1.5 mg/dl and creatinine≤2.0 mg/dl), moderate to severe valve disease, atrial fibrillation, severe arrhythmias, or diseases severely reducing the life expectancy or limiting study participation. |
| Jonker 2013^19^ | T2D, with and without insulin, age <70 years, no complications (retinopathy, neuropathy, or nephropathy), hypertension (SBP>165 mmHg and/or DBP>95 mmHg), BMI>35 kg/m2, smoking, known cardiac disease (abnormal ECG, cardiomyopathy, coronary or valvular disease), drugs, or contraindications for MRI. |

CAD-coronary artery disease, CVD-cardiovascular disease, LVEF-left ventricle ejection fraction, T2D-type 2 diabetes, BMI-body mass index.

1. Ouzzani M, Hammady H, Fedorowicz Z, Elmagarmid A. Rayyan—a web and mobile app for systematic reviews. *Syst Rev*. 2016;5(1):210. doi:10.1186/s13643-016-0384-4

2. McGavock J, Sandra Mandic, Richard Lewanczuk, et al. Cardiovascular adaptations to exercise training in postmenopausal women with type 2 diabetes mellitus. *Cardiovasc Diabetol*. 2004;22(11):685-686. doi:10.1002/clc.4960221102

3. Van Ryckeghem L, Keytsman C, De Brandt J, et al. Impact of continuous vs. interval training on oxygen extraction and cardiac function during exercise in type 2 diabetes mellitus. *Eur J Appl Physiol*. 2022;122(4):875-887. doi:10.1007/s00421-022-04884-9

4. Brassard P, Legault S, Garneau C, Bogaty P, Dumesnil JG, Poirier P. Normalization of Diastolic Dysfunction in Type 2 Diabetics after Exercise Training. *Med Sci Sports Exerc*. 2007;39(11):1896-1901. doi:10.1249/mss.0b013e318145b642

5. Loimaala A, Groundstroem K, Rinne M, Nenonen A, Huhtala H, Vuori I. Exercise training does not improve myocardial diastolic tissue velocities in Type 2 diabetes. *Cardiovasc Ultrasound*. 2007;5(1):32. doi:10.1186/1476-7120-5-32

6. Hordern MD, Coombes JS, Cooney LM, Jeffriess L, Prins JB, Marwick TH. Effects of exercise intervention on myocardial function in type 2 diabetes. *Heart*. 2009;95(16):1343-1349. doi:10.1136/hrt.2009.165571

7. Hare JL, Hordern MD, Leano R, Stanton T, Prins JB, Marwick TH. Application of an Exercise Intervention on the Evolution of Diastolic Dysfunction in Patients With Diabetes Mellitus: Efficacy and Effectiveness. *Circ Heart Fail*. 2011;4(4):441-449. doi:10.1161/CIRCHEARTFAILURE.110.959312

8. Schrauwen-Hinderling VB, Meex RC, Hesselink MK, et al. Cardiac lipid content is unresponsive to a physical activity training intervention in type 2 diabetic patients, despite improved ejection fraction. *Cardiovasc Diabetol*. 2011;10(1):47. doi:10.1186/1475-2840-10-47

9. Schmidt JF, Andersen TR, Horton J, et al. Soccer Training Improves Cardiac Function in Men with Type 2 Diabetes. *Med Sci Sports Exerc*. 2013;45(12):2223-2233. doi:10.1249/MSS.0b013e31829ab43c

10. Gulsin GS, Swarbrick DJ, Athithan L, et al. Effects of Low-Energy Diet or Exercise on Cardiovascular Function in Working-Age Adults With Type 2 Diabetes: A Prospective, Randomized, Open-Label, Blinded End Point Trial. *Diabetes Care*. 2020;43(6):1300-1310. doi:10.2337/dc20-0129

11. Wilson GA, Wilkins GT, Cotter JD, Lamberts RR, Lal S, Baldi JC. HIIT Improves Left Ventricular Exercise Response in Adults with Type 2 Diabetes. *Med Sci Sports Exerc*. 2019;51(6):1099-1105. doi:10.1249/MSS.0000000000001897

12. Suryanegara J, Cassidy S, Ninkovic V, et al. High intensity interval training protects the heart during increased metabolic demand in patients with type 2 diabetes: a randomised controlled trial. *Acta Diabetol*. 2019;56(3):321-329. doi:10.1007/s00592-018-1245-5

13. Cassidy S, Thoma C, Hallsworth K, et al. High intensity intermittent exercise improves cardiac structure and function and reduces liver fat in patients with type 2 diabetes: a randomised controlled trial. *Diabetologia*. 2016;59(1):56-66. doi:10.1007/s00125-015-3741-2

14. Heiskanen MA, Sjöros TJ, Heinonen IHA, et al. Sprint interval training decreases left-ventricular glucose uptake compared to moderate-intensity continuous training in subjects with type 2 diabetes or prediabetes. *Sci Rep*. 2017;7(1):10531. doi:10.1038/s41598-017-10931-9

15. Sacre JW, Jellis CL, Jenkins C, et al. A six-month exercise intervention in subclinical diabetic heart disease: Effects on exercise capacity, autonomic and myocardial function. *Metabolism*. 2014;63(9):1104-1114. doi:10.1016/j.metabol.2014.05.007

16. Hollekim-Strand SM, Bjørgaas MR, Albrektsen G, Tjønna AE, Wisløff U, Ingul CB. High-Intensity Interval Exercise Effectively Improves Cardiac Function in Patients With Type 2 Diabetes Mellitus and Diastolic Dysfunction. *J Am Coll Cardiol*. 2014;64(16):1758-1760. doi:10.1016/j.jacc.2014.07.971

17. Hollekim-Strand SM, Høydahl SF, Follestad T, et al. Exercise Training Normalizes Timing of Left Ventricular Untwist Rate, but Not Peak Untwist Rate, in Individuals with Type 2 Diabetes and Diastolic Dysfunction: A Pilot Study. *J Am Soc Echocardiogr*. 2016;29(5):421-430.e2. doi:10.1016/j.echo.2016.01.005

18. Cugusi L, Cadeddu C, Nocco S, et al. Effects of an Aquatic-Based Exercise Program to Improve Cardiometabolic Profile, Quality of Life, and Physical Activity Levels in Men With Type 2 Diabetes Mellitus. *PM&R*. 2015;7(2):141-148. doi:10.1016/j.pmrj.2014.09.004

19. Jonker JT, Snel M, Hammer S, et al. Sustained cardiac remodeling after a short-term very low calorie diet in type 2 diabetes mellitus patients. *Int J Cardiovasc Imaging*. 2014;30(1):121-127. doi:10.1007/s10554-013-0302-y
