# Supplementary material for: Effects of exercise on cardiac structure and function in patients with type 2 diabetes: a narrative review of prospective imaging studies
Source: Int J Cardiovasc Imaging. 2025 Jul 19;41(10):1851–64. doi: 10.1007/s10554-025-03457-z (PMC12491076; doi:10.1007/s10554-025-03457-z)
Supplement: Supplementary file 2 — Supplementary Material 2 [file 10554_2025_3457_MOESM2_ESM.docx]

Supplement 4. Effects of exercise on cardiac structure and function in type 2 diabetes

Table 1. Effects of exercise on the left ventricular structure in a supine position at rest

| Study | Group | Tool | IVSd  diastolic  (mm) | LVPWd  diastolic (mm) | LVPWd  systolic (mm) | LVDd  (mm) | LVSd  (mm) | Eccentricity  ratio (g/ml) | LVEDV (ml) | LVEDV/(BSA or FFM) (ml/m^2^ OR ml/kg) |
| --- | --- | --- | --- | --- | --- | --- | --- | --- | --- | --- |
| Van Ryckeghem 2022­­­(23) | MICT | echo | 11±1 | 11±2 |  | 40±3 |  |  |  |  |
|  | HIIT | echo | 12±1 | 12±2 |  | 45±4 |  |  |  |  |
| Brassard 2007(33) | MICT | echo | 9.9±1.1 | 9.4±0.8 |  | 49±3 | 30±4 |  |  |  |
|  | CON | echo | 10.1±1.7 | 10±2 |  | 50±2 | 30±4 |  |  |  |
| Hordern 2014 & Hare 2011(28,31) | MICT+RT+Diet | echo |  |  |  | 47±6 | 28±5 |  |  |  |
|  | CON | echo |  |  |  | 46±6 | 27±6 |  |  |  |
| Schrauwen- Hinderling 2011(35) | MICT+RT | CMR |  |  |  |  |  |  | 180[9] |  |
| Schmidt 2013(32) | SOCCER | echo |  |  |  | 50±1↑# |  |  | 119±6↑†# |  |
|  | CON | echo |  |  |  | 48±47 |  |  | 121±10 |  |
| Gulsin 2020(22) | MIT | Echo+CMR |  |  |  |  |  |  | 147(129,161) | 68(62,71) |
|  | CON | Echo+CMR |  |  |  |  |  |  | 134(125,149) | 63(58,68) |
| Wilson 2019(24) | HIIT | echo |  |  |  |  |  |  |  | 1.87[0.08]# |
|  | CON | echo |  |  |  |  |  |  |  | 2.00[0.16] |
| Cassidy 2016(34) | HIIT | CMR |  | 10.7±3.1 | 6.2±1.5 |  |  | 0.94±0.28 | 118±30↑# |  |
|  | CON | CMR |  | 9.1±2.5↑ | 5.5±1.1↑ |  |  | 0.85±0.24 | 129±28 |  |
| Heiskanen 2017(38) | SIT | CMR |  |  |  |  |  |  | 135(115,155)† | 67(60, 73)† |
|  | MICT | CMR |  |  |  |  |  |  | 156(139,173) | 76(71,82) |
| Cugusi 2015(36) | AQUATIC | echo |  |  |  |  |  |  | 79.4±17.8 |  |
| Jonker 2013(16) | MIT+RT+Trekking | echo |  |  |  |  |  |  | 176±9 |  |

Data are mean±SD, mean[SEM] or median(IQ) of baseline data. MICT-moderate-intense continuous training, HIIT-high-intense interval training, RT-resistance training, CON-control group, CMR-cardiac magnetic resonance imaging, CBIO-continuous bioreactance, IVSd-interventricular septal diameter diastolic, LVPWd-left ventricle posterior wall diameter, LVDd-left ventricle diastolic diameter, LVSd-left ventricle systolic diameter, LVEDV-left ventricle end-diastolic volume, ↑/↓- significant increase or decrease within group, †-significant time effect, #-significant between-group difference or interaction effect, p<0.05.

Table 2. The effects of exercise on the left atrial and ventricular structure in a supine position at rest

| Study | Group | Tool | LVESV  (ml) | LVESV/(BSA or FFM) (ml/m^2^ OR ml/kg) | LVM  (g) | LVMi (g/m² or g/ml($) | LVOT (mm) | LAV (mm) |
| --- | --- | --- | --- | --- | --- | --- | --- | --- |
| Van Ryckeghem 2022­­­(23) | MICT | echo |  |  | 142±34 | 71±14 | 21±1↑# |  |
|  | HIIT | echo |  |  | 190±48 | 88±16 | 22±1 |  |
| Brassard 2007(33) | MICT | echo |  |  | 88±13 |  |  | 39±4 |
|  | CON | echo |  |  | 92±12 |  |  | 40±2 |
| Hordern 2014 & Hare 2011(28,31) | MICT+RT+Diet | echo |  |  | 203±66 | 97±26 |  |  |
|  | CON | echo |  |  | 206±62 | 99±28 |  |  |
| Schrauwen-Hinderling 2011(35) | MICT+RT | CMR | 90[7]↓ |  |  |  |  |  |
| Schmidt 2013(32) | SOCCER | echo |  |  |  | 79±3.8↑# |  |  |
|  | CON | echo |  |  |  | 70.3±3.4 |  |  |
| Gulsin 2020(22) | MIT | Echo+CMR |  |  | 123±22 | 0.86(0.75-0.91) |  |  |
|  | CON | Echo+CMR |  |  | 116±23 | 0.82(0.77-0.86) |  |  |
| Wilson 2019(24) | HIIT | echo |  | 0.77[0.06] |  | 3.37[0.19] |  |  |
|  | CON | echo |  | 0.75[0.06] |  | 3.54[0.91] |  |  |
| Cassidy 2016(34) | HIIT | CMR | 42±17 |  | 104±17↑# |  |  |  |
|  | CON | CMR | 50±22 |  | 107±25 |  |  |  |
| Heiskanen 2017(38) | SIT | CMR | 48(36,60)† | 24(19,28)† | 100(86,113)† | 49(44,54)† |  |  |
|  | MICT | CMR | 56(46,66) | 27(23,31) | 120(109,132) | 59(55,63) |  |  |
| Sacre 2014(37) | MIT+RT+HOME | echo |  |  |  | 79(5) |  |  |
|  | CON | echo |  |  |  | 78(4) |  |  |
| Cugusi 2015(36) | AQUATIC | echo | 29.4±8.3 |  |  |  |  |  |
| Jonker 2013(16) | MIT+RT+Trekking | echo | 77[4] |  | 103[8] | 50[3] |  |  |

Data are mean±SD, mean[SEM] or median(IQ) of baseline data. MICT-moderate-intense continuous training, HIIT-high-intense interval training, AT-moderate intense training, RT-resistance training, CON-control group, CMR-cardiac magnetic resonance imaging, LVESV-left ventricle end-systolic volume, BSA-body surface area, LVM-left ventricle mass, LVMi-left ventricle mass per body surface area or volume, LVOT-left ventricle outflow tract diameter, LAV-left atrial volume, ↑/↓- significant increase or decrease within group, †-significant time effect, #-significant between-group difference or interaction effect, p<0.05.

Table 3. The effects of exercise on the right ventricular structure in a supine position at rest

| Study | Group | Tool | RVmass  (g) | RVmass/BSA  (g/m^2^) | RVEDV  (ml) | RVEDV/BSA  (ml/m^2^) | RVESV  (ml) | RVESV/BSA  (ml/m^2^) | Calibrated  integrated  backscatter  (dB) |
| --- | --- | --- | --- | --- | --- | --- | --- | --- | --- |
| Heiskanen 2017(38) | SIT | CMR | 25(22,28)†# | 12(11,13)†# | 145(126,165)†# | 72(66,78)†# | 59(46,72)† | 29(24,34)† |  |
|  | MICT | CMR | 28(25,30) | 13(13,14) | 167(151,184) | 82(76,87) | 69(58,79) | 33(29,38) |  |
| Sacre 2014(37) | MIT+RT+HOME | echo |  |  |  |  |  |  | -16.2(1.0)↓ |
|  | CON | echo |  |  |  |  |  |  | -16.7(0.9) |

Data are mean±SD, mean[SEM] or median(IQ) of baseline data. CON-control, MICT-moderate intense continuous training, SIT-sprint interval training, MIT-moderate intense training, RT-resistance training, CMR-cardiac magnetic resonance imaging, echo-echocardiography, RV-right ventricle, EDV-end-diastolic volume, ESV-end-systolic volume, BSA-body surface area, ↑/↓- significant increase or decrease within group, †-significant time effect, #-significant between-group difference or interaction effect, p<0.05.

Table 4. The effects of exercise on the left ventricular systolic function in a supine position at rest

| Study | Group | Tool | s’ pwTDI  (cm/s) | s’ cTDI  (cm/s) | IVCT  (m/s) | ET  (m/s) | LVEF  (%) | LVSV  (ml) | LVSV/FFM  (ml/kg) | LVSV/BSA  (ml/m^2^) |
| --- | --- | --- | --- | --- | --- | --- | --- | --- | --- | --- |
| Van Ryckeghem 2022­­­(23) | MICT | echo | 7±1 |  | 70±15 | 271±17↑ |  |  |  |  |
|  | HIIT | echo | 7±2 |  | 80±27 | 279±31 |  |  |  |  |
| Brassard 2007(33) | MICT | echo |  |  |  |  | 66±4 |  |  |  |
|  | CON | echo |  |  |  |  | 66±5 |  |  |  |
| Loimaala 2007(40) | MICT+RT | echo | graph |  |  |  |  |  |  |  |
|  | CON | echo | graph |  |  |  |  |  |  |  |
| Hordern 2014 & Hare 2011(28,31) | MICT+RT+Diet | echo | 5.3±1.3↑ |  |  |  | 71±7 |  |  |  |
|  | CON | echo | 5.2±0.8↑ |  |  |  | 72±8 |  |  |  |
| Schrauwen-Hinderling 2011(35) | MICT+RT | CMR |  |  |  |  | 51[2]↑ |  |  |  |
| Schmidt 2013(32) | SOCCER | echo | 8±1 | 6.1±0.4↑† |  |  | 58.1±1.0† |  |  |  |
|  | CON | echo | 8±0 | 6.0±0.4 |  |  | 58.1±1.1 |  |  |  |
| Gulsin 2020(22) | MIT | Echo+CMR |  |  |  |  | 66.8±7.9 |  |  |  |
|  | CON | Echo+CMR |  |  |  |  | 67.6±5.4 |  |  |  |
| Wilson 2019(24) | HIIT | echo | 8[0] |  |  |  | 59[2] |  | 1.11[0.06]# |  |
|  | CON | echo | 7[0] |  |  |  | 62[2] |  | 1.24[0.11] |  |
| Suryanegara 2019(25) | HIIT | CBIM |  |  |  |  |  | 78±15 |  |  |
|  | CON | CBIM |  |  |  |  |  | 82±15 |  |  |
| Cassidy 2016(34) | HIIT | CMR |  |  |  |  | 65±8 | 76±16↑ |  |  |
|  | CON | CMR |  |  |  |  | 64±11 | 79±14 |  |  |
| Heiskanen 2017(38) | SIT | CMR |  |  |  |  | 65(62,69) | 87(75,98) |  | 43(39,47)# |
|  | MICT | CMR |  |  |  |  | 64(61,68) | 100(90,109) |  | 49(46,52) |
| Sacre 2014(37) | MIT+RT+HOME | echo |  | 5.3[0.2] |  |  | 62[1] |  |  |  |
|  | CON | echo |  | 5.8[0.2] |  |  | 65[1] |  |  |  |
| Hollekim-Strand 2014-16(29,30) | Home MIT | echo | 7.7±12 |  |  |  |  |  |  |  |
|  | HIIT | echo | 6.8±0.8↑# |  |  |  |  |  |  |  |
| Cugusi 2015(36) | AQUATIC | echo | 6.92±0.86 |  |  |  | 65±6 |  |  |  |
| Jonker 2013(16) | MIT+RT+Trekk | echo |  |  |  |  | 56[1] | 99[5] |  |  |

Data are mean±SD, mean[SEM] or median(IQ) of baseline data. Trekk-Trekking, HIIT-high intense interval training, CON-control, MICT-moderate intense continuous training, MIT-moderate intense training, RT-resistance training, CMR-cardiac magnetic resonance imaging, echo-echocardiography, CBIM-continuous bioreactance method, s’-mitral annulus systolic velocity, c- or pw-TDI=colored or pulsed wave tissue doppler imaging, IVCT-isovolumetric contraction time, ET-ejection time, LVEF-left ventricle ejection fraction, LVSV-left ventricle stroke volume, FFM-fat free mass, BSA-body surface area, ↑/↓- significant increase or decrease within group, †-significant time effect, #-significant between-group difference or interaction effect, p<0.05.

Table 5. The effects of exercise on the left and right ventricular systolic function in a supine position at rest

| Study | Group | Tool | LVCO  (l/min) | LVCO/(BSA or FFM)  (l/min/(m^2^ or kg) | SW  (ml/mmHg) | LVdis  (mm) | RVSV  (ml) | RVSV/BSA  (ml/m^2^) | RVCO  (l/min) | RVEF  (%) |
| --- | --- | --- | --- | --- | --- | --- | --- | --- | --- | --- |
| Schrauwen-Hinderling 2011(35) | MICT+RT | CMR | 5.08[0.3]↑ | 2.87[1.61]↑ |  |  |  |  |  |  |
| Schmidt 2013(32) | SOCCER | echo |  |  |  | 9.7±0.7↑ |  |  |  |  |
|  | CON | echo |  |  |  | 10.6±0.8 |  |  |  |  |
| Wilson 2019(24) | HIIT | echo |  | 0.09[0.01] | 92[8]† |  |  |  |  |  |
|  | CON | echo |  | 0.09[0.00] | 83[8] |  |  |  |  |  |
| Suryanegara 2019(25) | HIIT | CBIM | 6.1±1.2 |  |  |  |  |  |  |  |
|  | CON | CBIM | 6.5±2.6 |  |  |  |  |  |  |  |
| Cassidy 2016(34) | HIIT | CMR | 5.0±1.1 |  |  |  |  |  |  |  |
|  | CON | CMR | 5.0±1.0 |  |  |  |  |  |  |  |
| Heiskanen 2017(38) | SIT | CMR | 6.3(5.6,7.0) | 3.2(2.9,3.5) |  |  | 86(76,97) | 43(39,47) | 6.2(5.6,6.9) | 60(56,64)† |
|  | MICT | CMR | 6.8(6.2,7.4) | 3.2(2.9,3.5) |  |  | 99(90,108) | 48(45,51) | 6.7(6.2,7.3) | 60(56,63) |
| Jonker 2013(16) | MIT+RT+Trekking | echo |  | 3.1±0.1 |  |  |  |  |  |  |

Data are mean±SD, mean[SEM] or median(IQ) of baseline data. HIIT-high intense interval training, CON-control, MICT-moderate intense continuous training, SIT-sprint interval training, CMR-cardiac magnetic resonance imaging, echo-echocardiography, CBIM-continuous bioreactance method, LV-left ventricle, CO-cardiac output, BSA-body surface area, FFM-fat free mass, SW-stroke work, LVdis-left ventricle displacement, RVSV-right ventricle stroke volume, RVCO-right ventricle cardiac output, RVEF-right ventricle ejection fraction, ↑/↓- significant increase or decrease within group, †-significant time effect, #-significant between-group difference or interaction effect, p<0.05.

Table 6. The effects of exercise on the left and right ventricular systolic function in a supine position at rest

| Study | Group | Tool | TAPSE  (cm) | Global  Strain (%) | Strain rate (cm/s) | Strain rate  (1/Strain) | Peak endocardial  circumferential strain (%) | Peak whole wall circumferential  strain (%) | Peak  twist  (°) |
| --- | --- | --- | --- | --- | --- | --- | --- | --- | --- |
| Loimaala 2007(40) | MICT+RT | echo |  | graph |  | graph |  |  |  |
|  | CON | echo |  |  |  |  |  |  |  |
| Hordern 2014 & | MICT+RT+Diet | echo |  | 20.6±3.6↑ |  | 1.4±0.3 |  |  |  |
| Hare 2011(28,31) | CON | echo |  | 20.5±4.1↑ |  | 1.4±0.3 |  |  |  |
| Schmidt 2013(32) | SOCCER | echo | 2.1±0.1↑†# | 15.5±0.9↑# |  |  |  |  |  |
|  | CON | echo | 2.2±0.1 | 17.5±1.0↑ |  |  |  |  |  |
| Gulsin 2020(22) | MIT | echo+CMR |  | 16.3±2.9 |  | 0.92±0.20↑ |  |  |  |
|  | CON | echo+CMR |  | 17.4±2.2 |  | 1.06±0.15 |  |  |  |
| Cassidy 2016(34) | HIIT | CMR |  | 12.2±3.0 |  |  | 25.2±4.6 | 16.5±3.1 | 8.1±1.8↓# |
|  | CON | CMR |  | 13.1±2.2 |  |  | 23.1±4.1 | 16.5±31 | 7.1±2.2 |
| Sacre 2014(37) | MIT+RT+HOME | echo |  | 20.8(0.7)↑ |  | 1.3(0.1)↑ |  |  |  |
|  | CON | echo |  | 21.0(0.7)↑ |  | 1.4(0.1)↑ |  |  |  |
| Hollekim-Strand | Home MIT | echo |  | 16.7±2.2 |  | 1.0±0.15 |  |  | 12±5.7 |
| 2014-16(29,30) | HIIT | echo |  | 17.2±1.9↑ |  | 0.87±0.11↑# |  |  | 12.2±4.7 |
| Cugusi 2015(36) | AQUATIC | echo |  |  | 12.1±3.0 | 0.67±0.07 |  |  |  |

Data are mean±SD, mean[SEM] or median(IQ) of baseline data. HIIT-high intense interval training, CON-control, MICT-moderate intense continuous training, MIT-moderate intense training, RT-resistance training, echo-echocardiography, CMR-cardiac magnetic resonance imaging, TAPSE-tricuspid annular plane systolic excursion, graph-data graphically shown,↑/↓- significant increase or decrease within group, †-significant time effect, #-significant between-group difference or interaction effect, p<0.05.

Table 7. The effects of exercise on the left ventricular systolic function in a supine position at rest

| Study | Group | Tool | Peak LV  basal rotation  (°) | LV basal  twist rate  (°/s) | LV basal  untwist rate  (°/s) | Time to  peak basal  untwist rate  (%diastole) | Peak LV  apical  rotation  (°) | LV apical  twist rate  (°/s) | LV apical  untwist rate  (°/s) | Time to  peak apical  untwist rate  (%diastole) |
| --- | --- | --- | --- | --- | --- | --- | --- | --- | --- | --- |
| Hollekim-Strand 2014-16(29,30) | Home MIT | echo | -5.3±3.8 | -54.4±24.8 | 60.9±29.6 | 14.4±6↓ | 8.9±3.7 | 52±29.6 | -56.9±28.1 | 21±9.2↓ |
|  | HIIT | echo | -5.6±2.7 | -54.1±27.9 | 54±21.8 | 13.5±8.3↓ | 8±3.6 | 46.9±18.3 | -62.5±23 | 18.1±9.6↓ |

Data are mean±SD, mean[SEM] or median(IQ) of baseline data. HIIT-high intense interval training, MIT-moderate intense training, echo-echocardiography, LV-left ventricle, ↑/↓- significant increase or decrease within group, †-significant time effect, #-significant between-group difference or interaction effect, p<0.05.

Table 8. The effects of exercise on the left ventricular twist and untwist supine at rest

| Study | Group | Tool | Peak LV twist rate (°/s) | Peak LV untwist rate (°/s) | Time to peak untwist rate (%diastole) |
| --- | --- | --- | --- | --- | --- |
| Hollekim-Strand 2014-16(29,30) | Home MIT | echo | 72±33.8 | -86.5±34.9 | 16.1±9↓ |
|  | HIIT | echo | 73.3±30.9 | -80.2±32.6 | 16.5±9↓ |

Data are mean±SD, mean[SEM] or median(IQ) of baseline data. HIIT-high intense interval training, MIT-moderate intense training, echo-echocardiography, LV-left ventricle, ↑/↓- significant increase or decrease within group, †-significant time effect, #-significant between-group difference or interaction effect, p<0.05.

Table 9. The effects of exercise on the left ventricular systolic function in semi-supine at rest, low-intense exercise (40%VO2max), moderate-intense exercise (60%VO2max) and peak exercise

| Study | Group | Tool | Condition | LVEF  (%) | SW  (ml/mmHg) | LVCO  (L/min) | CI  (L/min/m^2^) | Global strain  (%) | ∆Global strain  (rest-peak, %) |
| --- | --- | --- | --- | --- | --- | --- | --- | --- | --- |
| Van Ryckeghem 2022­­­(23) | MICT | CPETecho | rest |  |  | 4.7±0.9 | 2.38±0.4 | 17.3±1.6 |  |
|  | HIIT | CPETecho | rest |  |  | 5±1.1 | 2.35±0.52 | 16.6±2.8 |  |
|  | MICT | CPETecho | peak exercise |  |  | 11.4±2.3 | 5.7±1.06 | 21.1±3 | 4.6±3.1 |
|  | HIIT | CPETecho | peak exercise |  |  | 13.7±2.3 | 6.44±1.35 | 20.2±3.6 | 4.4±3 |
| Wilson 2019(24) | HIIT | CPETecho | rest | 54.8[2.3]†# | 89.0[6.8]# |  |  |  |  |
|  | CON | CPETecho | rest | 62.7[1.5] | 87.7[5.9] |  |  |  |  |
|  | HIIT | CPETecho | low-intense exercise | 56.6[3.0]†# | 88.6[7.8]# |  |  |  |  |
|  | CON | CPETecho | low-intense exercise | 69.6[3.2] | 103.8[13.9] |  |  |  |  |
|  | HIIT | CPETecho | moderate-intense exercise | 61.9[2.4]†# | 106.7[5.1]† |  |  |  |  |
|  | CON | CPETecho | moderate-intense exercise | 73.3[1.9] | 123.3[6.1] |  |  |  |  |

Data are mean±SD, mean[SEM] or median(IQ) of baseline data. HIIT-high intense interval training, CON-control, MICT-moderate-intense continuous training, LVEF-left ventricle ejection fraction, SW-stroke work, LVCO-cardiac output, CI-cardiac index, CPETecho-cardiopulmonary exercise testing with echocardiography, peak exercise-exercise at respiratory exchange ratio >1.03, ↑/↓- significant increase or decrease within group, †-significant time effect, #-significant between-group difference or interaction effect, p<0.05.

Table 10. The effects of exercise on diastolic function of the left atrium and ventricle in a supine position at rest

| Study | Group | Tool | E  (cm/s) | e’ pwTDI  (cm/s) | e’ colorTDI  (cm/s) | Early LV  filling rate (ml/s) | Early LV  filling (%) | E/e’ |
| --- | --- | --- | --- | --- | --- | --- | --- | --- |
| McGavock 2004(39) | CON | echo | 97±23 |  |  |  |  |  |
|  | MICT+RT | echo | 87±10 |  |  |  |  |  |
| Van Ryckeghem 2022­­­(23) | MICT | echo | 63±15 | 6±2 |  |  |  | 12±2.3 |
|  | HIIT | echo | 54±10 | 5±1 |  |  |  | 10.1±1.8 |
| Brassard 2007(33) | MICT | echo | 60±12 |  |  |  |  |  |
|  | CON | echo | 68±15 |  |  |  |  |  |
| Loimaala 2007(40) | MICT+RT | echo | 64[3] |  |  |  |  | 9.0 |
|  | CON | echo | 69(3) |  |  |  |  | 8.5 |
| Hordern 2014 & Hare 2011(28,31) | MICT+RT+Diet | echo |  | 5.5±1.7↑ |  |  |  |  |
|  | CON | echo |  | 5.8±2.1↑ |  |  |  |  |
| Schmidt 2013(32) | SOCCER | echo |  | 7±1↑# | 6.0±0.7↑†# |  |  | 9.7±1.2↑ |
|  | CON | echo |  | 10±0 | 7.8±0.6 |  |  | 6.8±0.5 |
| Gulsin 2020(22) | MIT | echo+CMR |  |  |  |  |  | 8.8 (7.0,10.6) |
|  | CON | echo+CMR |  |  |  |  |  | 8.0 (6.5,9.7) |
| Wilson 2019(24) | HIIT | echo | 63[4] | 8[1] |  |  |  | 8.5[0.6] |
|  | CON | echo | 64[6] | 6[0] |  |  |  | 9.6[0.5] |
| Cassidy 2016(34) | HIIT | CMR |  |  |  | 241±84↑# | 57±9↑ |  |
|  | CON | CMR |  |  |  | 250±44 | 58±11 |  |
| Sacre 2014(37) | MIT+RT+HOME | echo |  |  | 4.7[0.2]↑ |  |  | 13.4[0.8]↑ |
|  | CON | echo |  |  | 5.0 [0.2]↑ |  |  | 11.8[0.8] |
| Hollekim-Strand 2014-16(29,30) | Home MIT | echo | 63.4±9.5#† | 7.1±0.7↑# |  |  |  | 9.1±1.8 |
|  | HIIT | echo | 64.8±10.7↑ | 7.0±0.7↑ |  |  |  | 9.3±1.7 |
| Cugusi 2015(36) | AQUATIC | echo |  |  |  |  |  | 10.1±1.5↓ |
| Jonker 2013(16) | MIT+RT+Trekking | echo |  |  |  |  |  | 10.8[0.64] |

Data are mean±SD, mean[SEM] or median(IQ) of baseline data. MICT-moderate-intense continuous training, HIIT-high-intense interval training, MIT-moderate intense training, RT-resistance training, CON-control group, CMR-cardiac magnetic resonance imaging, CBIM-continuous bioreactance, E-early inflow velocity at mitral annulus, e’pwTDI-early tissue velocity at mitral annulus via pulsed wave tissue doppler imaging, e’colorTDI-early tissue velocity at mitral annulus via colored tissue doppler imaging, ↑/↓- significant increase or decrease within group, †-significant time effect, #-significant between-group difference or interaction effect, p<0.05. Data are supine at rest unless otherwise indicated.

Table 11. The effects of exercise on diastolic function of the left atrium and ventricle in a supine position at rest

| Study | Group | Tool | A  (cm/s) | a’  (cm/sec) | Late LV filling rate  (ml/s) | E/A | E-deceleration  time (ms) | E-deceleration  peak (ml/ms) | IVRT  (ms) |
| --- | --- | --- | --- | --- | --- | --- | --- | --- | --- |
| McGavock 2004(39) | CON | echo | 93±11 |  |  | 1.0±0.2 | 206±29 |  |  |
|  | MICT+RT | echo | 95±29 |  |  | 1.0±0.3 | 256±39 |  |  |
| Van Ryckeghem 2022­­­(23) | MICT | echo | 86±17 | 10±2 |  | 0.77±0.24 | 194±56 |  | 89±32 |
|  | HIIT | echo | 65±10 | 9±2 |  | 0.84±0.17 | 208±24 |  | 102±25 |
| Brassard 2007(33) | MICT | echo | 63±13↓ |  |  | 0.76±0.11↑ | 209±36 |  | 112±10 |
|  | CON | echo | 72±16 |  |  | 0.78±0.09 | 221±49 |  | 103±13↑ |
| Loimaala 2007(40) | MICT+RT | echo | 68(3) |  |  |  |  |  |  |
|  | CON | echo | 66(3) |  |  |  |  |  |  |
| Schmidt 2013(32) | SOCCER | echo |  |  |  | 0.9±0.1↑# | 215±15↓# |  |  |
|  | CON | echo |  |  |  | 1.2±0.1 | 176±8 |  |  |
| Gulsin 2020(22) | MIT | echo+CMR |  |  |  | 0.94±0.19 |  |  |  |
|  | CON | echo+CMR |  |  |  | 1.00±0.21 |  |  |  |
| Wilson 2019(24) | HIIT | echo | 70[4] |  |  | 0.93[0.08] |  |  |  |
|  | CON | echo | 70[7] |  |  | 0.98[0.16] |  |  |  |
| Cassidy 2016(34) | HIIT | CMR |  |  | 278±67 |  |  |  |  |
|  | CON | CMR |  |  | 310±143 |  |  |  |  |
| Sacre 2014(37) | MIT+RT+HOME | echo |  |  |  | 0.89 [0.04] | 232[11] |  |  |
|  | CON | echo |  |  |  | 0.77 [0.04]↑ | 232[11] |  |  |
| Hollekim-Strand 2014-16 (29,30) | Home MIT | echo |  |  |  | 0.92±0.18†# |  |  |  |
|  | HIIT | echo |  |  |  | 0.93±0.21↑ |  |  |  |
| Jonker 2013(16) | MIT+RT+Trekking | echo |  |  |  | 1.45[0.16] |  | 3.9[4.3] |  |

Data are mean±SD, mean[SEM] or median(IQ) of baseline data. MICT-moderate-intense continuous training, HIIT-high-intense interval training, MIT-moderate intense training, RT-resistance training, CON-control group, CMR-cardiac magnetic resonance imaging, CBIM-continuous bioreactance method, A-late inflow velocity at the mitral annulus, a’-late tissue velocity at mitral annulus, LV-left ventricle, IVRT-isovolumetric relaxation time, ↑/↓- significant increase or decrease within group, †-significant time effect, #-significant between-group difference or interaction effect, p<0.05.

Table 12. The effects of exercise on diastolic function of the left atrium and ventricle in a supine position at rest

| Study | Group | Tool | LVPEDSR (1/s) | Myocardial perfusion  Reserve | Aortic distensibility (1/mmHg x 0.01) | Pva (m/s) | Pvs/Pvd (m/s) |
| --- | --- | --- | --- | --- | --- | --- | --- |
| McGavock 2004(39) | CON | echo |  |  |  | 28±4 | 1.4±0.2 |
|  | MICT+RT | echo |  |  |  | 28±7 | 1.5±0.1 |
| Gulsin 2020(22) | MIT | echo+CMR | 0.92±0.20# | 3.3±0.9 | 3.3(2.7–5.7) |  |  |
|  | CON | echo+CMR | 1.06±0.15 | 2.7±0.8 | 3.7(2.9–5.5) |  |  |

Data are mean±SD, mean[SEM] or median(IQ) of baseline data. MIT-moderate-intense training, CON-control group, RT-resistance training, CMR-cardiac magnetic resonance imaging,

echo-echocardiography, LVPEDSR-left ventricular peak early diastolic strain rate, Pva-pulmonary venous flow during atrial systole, Pvs/Pvd- pulmonary venous flow in systole by pulmonary venous flow in diastole, ↑/↓- significant increase or decrease within group, †-significant time effect, #-significant between-group difference or interaction effect, p<0.05.

Table 13. The effects of exercise on diastolic function in semi-supine position at rest and peak exercise

| Study | Group | Tool | E  (cm/s) | e’ pwTDI  (cm/s) | E/e’ |
| --- | --- | --- | --- | --- | --- |
| Van Ryckeghem 2022­­­(23) | MICT | echo rest | 62±14 | 6±1 | 11.4 ± 3.9 |
|  | HIIT | echo rest | 57±18 | 6±1 | 9.8 ± 2.7 |
|  | MICT | echo peak exercise | 106±24 | 11±5 | 10.9±5 |
|  | HIIT | echo peak exercise | 123±10 | 14±5 | 10.3±5.9↓ |

Data are mean±SD of baseline data, MICT-moderate-intense continuous training, HIIT-high-intense interval training, E-early mitral annulus inflow velocity, e’pwTDI-early mitral annulus tissue velocity via pulsed wave tissue doppler imaging, ↑/↓- significant increase or decrease within group, †-significant time effect, #-significant between-group difference or interaction effect, p<0.05.
